# The functional roles of deoxyelephantopin potential target circTNPO3 in regulating pancreatic cancer malignant phenotype and gemcitabine chemoresistance via miR-188-5p/CDCA3/TRAF2-mediated remodeling of NF-κB signaling pathway

**DOI:** 10.3389/fphar.2025.1613560

**Published:** 2025-07-31

**Authors:** Daolin Ji, Jia Liu, Yanhui Zhang, Li Hou, Haonan Feng, Xue Xing, Deming Guan, Tiangang Cui, Yi Xu, Gang Tan

**Affiliations:** ^1^ Department of Hepatopancreatobiliary Surgery, The Fourth Affiliated Hospital, Harbin Medical University, Harbin, China; ^2^ The Key Laboratory of Myocardial Ischemia, Harbin Medical University, Ministry of Education, Harbin, China; ^3^ Future Medical Laboratory, The Second Affiliated Hospital, Harbin Medical University, Harbin, China; ^4^ Department of Hepatopancreatobiliary Surgery, The Second Affiliated Hospital, Harbin Medical University, Harbin, China; ^5^ Key Laboratory of Human Development and Disease Research, Guangxi Medical University, Education Department of Guangxi Zhuang Autonomous Region, Nanning, Guangxi, China; ^6^ Department of Pathology, Li Ka Shing Faculty of Medicine, The University of Hong Kong, Pokfulam, Hong Kong SAR, China

**Keywords:** pancreatic cancer, circTNPO3, chemoresistance, stemness, deoxyelephantopin, gemcitabine, NF-κB signaling pathway

## Abstract

**Background:**

Pancreatic cancer (PC) has been one of the most severe digestive system malignant tumor with poor prognosis that threatens human health. Chemotherapy is essential for patients with advanced PC, but unfortunately the curative effect is limited by chemoresistance. CircTNPO3, a recently discovered circular RNA (circRNA), has been indicated to be associated with multi-types of tumors. However, the function and mechanism of circTNPO3 in regulating PC malignant phenotype and chemoresistance still remain obscure.

**Methods:**

qRT-PCR and ISH were used to analyze circTNPO3 expression in PC cells and pathological specimens. The subcellular localization of circTNPO3 was visualized through nucleoplasmic RNA separation and FISH assays. The effect of cicTNPO3 on PC cell proliferation, migration and invasion was assessed using EdU, colony formation, wound healing and Transwell assays respectively. Cell apoptosis was detected using ELISA, AO/EB, Hoechst 33342 and flow cytometry assays. The binding potential between circTNPO3, miR-188-5p and CDCA3 was verified by Ago2-RIP, RNA pull down and dual-luciferase reporter assays. The relationship between CDCA3, TRAF2 and NF-κB-p65 was analyzed using Pearson correlation, and the expression was detected using immunoblotting. The nucleus translocation of p65 was evaluated using IF assay. The effect of circTNPO3 on PC growth and metastasis was analyzed using subcutaneous and lung metastatic tumor models *in vitro*. Deoxyelephantopin, a small molecule extract from traditional Chinese medicine, was applied to evaluate the potential of circTNPO3 as therapeutic target.

**Results:**

CircTNPO3 was aberrantly highly expressed in PC cells and tissues, and negatively associated with patient prognosis and gemcitabine chemotherapy sensitivity. Functionally, silencing circTNPO3 attenuated the malignant phenotypes and chemoresistance of PC *in vitro* and *in vivo*, conversely, facilitated by circTNPO3 overexpression. Mechanically, cytoplasmic circTNPO3 functioned as a sponge of miR-188-5p, and partially alleviated the effect of miR-188-5p on downstream molecules, which further upregulate the CDCA3 and TRAF2 expression and NF-κB activity, finally promoted PC progression and chemoresistance. More innovatively, the potential of circTNPO3 as a novel diagnostic biomarker and therapeutic target for PC was primarily validated in present study.

**Conclusion:**

CircTNPO3 acted as an oncogenic and chemoresistant gene in PC, mechanically through targeting miR-188-5p and regulating CDCA3, TRAF2 and NF-κB signaling pathway.

## 1 Introduction

Pancreatic cancer (PC), which is the most lethal digestive system malignant tumor, has become a severe threat to human life and health worldwide (Mohamed et al., 2025). According to the recent survey report from Global Burden of Disease (GBD), the morbidity and mortality of PC, as well as the Disability-Adjusted Life Years (DALYs), have risen almost double from 1990 to 2019 (Li et al., 2024). Integrating analysis of global disease data from the International Agency for Research on Cancer (IARC) and GLOBOCAN, there were an estimated 510,566 new cases and 467,005 deaths of PC worldwide in 2022, accounting for 2.6% and 4.8% of total new cancer diagnoses and cancer-related deaths in the same year (Bray et al., 2024). As of 2022, numerous statistics suggest that PC has ranked the 10th and seventh most common new cancer cases in male and female, respectively (Siegel et al., 2025). It is postulated that by 2030 PC might soar into the second leading cause of cancer-related death in the United States, meanwhile, the seventh leading cause in Europe (Danpanichkul et al., 2024). Despite the best diagnostic and therapeutic strategies, the 5-year overall survival for PC still remains dismal, just approximately 8% (Kane et al., 2024). At present, surgical extirpation still remains the only existing potential method to heal PC, and it is also the foundation for comprehensive treatment (Wenzel et al., 2024). Due to the concealed pathogenesis and rapidly progressing, most sufferers have entered the advanced stages of PC until definitive diagnosis, costing the opportunity for surgical resection (Gao et al., 2024). Accumulative studies have shown that neoadjuvant chemotherapy could achieve the goal of tumor reduction and further confer the surgery opportunity for patients with borderline resectable, locally advanced and distant metastatic PC (Wu et al., 2024). Currently, studies around the theme of chemotherapy agents and regimens emerged in large numbers, including the representative gemcitabine (GEM), GEM plus nanoparticle albumin–bound paclitaxel (Nab-P), FOLFIRINOX (Fluorouracil, Leucovorin, Irinotecan, Oxaliplatin) and the derived modified (mFOLFIRINOX) regimens (Dekker et al., 2024). Given the unwieldy chemotherapy side-effects and limited benefits of FOLFIRINOX and mFOLFIRINOX regimens, GEM and its offspring scheme still remain the indispensable part of chemotherapy for metastatic PC (Goetze et al., 2024). However, it is regrettable that the intrinsic and acquired drug resistance, as well as the chemotherapy interruption caused by adverse drug reactions, dent the effectiveness of GEM in PC treatment (Shah et al., 2024; Younes et al., 2024). More realistically, the mysterious mechanism of PC progression and GEM chemoresistance is urgently needed to be explored and revealed to improve patient survival time and life quality.

With the development of network pharmacology, systematic pharmacology and traditional Chinese medicine (TCM) theoretical research, the pharmacological properties of TCM are gradually revealed (Zhang P. et al., 2023). TCM, especially traditional herbal medicine, characterized by multi-components and multi-targets, has been shown to be advantageous in addressing the complexity of malignant tumors, in which researches have primarily focused on TCM monomers (Li et al., 2025). Accumulative research have found that TCM not only could alleviate the symptoms of patients, improve their quality of life and prolong overall survival time, but also could reduce the adverse reactions and drug resistance caused by conventional chemotherapy, radiotherapy, immunotherapy or targeted therapy (Li et al., 2025). Among them, deoxyelephantopin (DET), a sesquiterpene lactone purified from the Chinese herbal medicine *Elephantopus scaber* L., has been confirmed to hold the antitumor potential in several malignancies, such as hepatocellular carcinoma, colorectal cancer and osteosarcoma (Mehmood and Muanprasat, 2022). Through comprehensively analyzing the application of *E. scaber* L. in the treatment of type II diabetes in TCM, as well as the issue that type II diabetes is a high-risk factor for PC and the idea of “preventive treatment for disease” in TCM theory, the antitumor effect of DET on PC has been investigated by our team. At present, the only two original studies on DET in PC were both conducted by our research colleague, finding that DET could effectively inhibit the malignant biological behavior of PC and improve chemoresistance to GEM, additionally, the prophylactic therapeutic efficacy have also been verified (Ji et al., 2020; Ji et al., 2022). Taking into account the lack of detailed mechanism is an inescapable issue that restricts the application of TCM in clinical practice, the antitumor molecular mechanism of DET in PC needs to be further elucidated.

Circular RNA (circRNA), which is recognized with closed-loop formula structure and 200 nt ∼2,000 nt length, is a special type of non-coding RNA (ncRNA) (Hwang and Kim, 2024). This unique pattern has been confirmed to be formed by splicing the 5′ end of the upstream exon with the 3′ end of the downstream exon (Hwang and Kim, 2024). Unlike traditional liner RNA, the most striking characteristic of circRNA is insusceptible with RNase R, possessing higher stability (Hwang and Kim, 2024). Despite the lack of direct protein-coding capacity, circRNA plays a central role in multiple cell biological processes and diseases, especially in human malignancies (Wei et al., 2025). Mechanistically, similar to long non-coding RNA (lncRNA), circRNA could also serve as competitive endogenous RNA (ceRNA) to entrap miRNA predatorily, regulating the function of target miRNA and downstream protein expression (Shahpari et al., 2024). Various medical studies have testified that aberrant versions of circRNAs are implicated in tumorigenesis, angiogenesis, metastasis and chemoresistance through complex molecular networks (Conn et al., 2024). Recent research has revealed a variety of circRNAs involved in PC progression and GEM chemoresistance, such as circMBOAT2 facilitates malignant biological behavior of PC through glutamine catabolism that is dependent on miR-433-3p/GOT1 axis (Zhou et al., 2021); CircFARP1 promotes GEM chemoresistance via cancer-associated fibroblasts associated with LIF/STAT3 axis in PC (Hu et al., 2022); CircCGNL1 suppresses PC progression via upregulating NUDT4/HDAC4/RUNX2/GAMT mediated apoptosis (Yuan et al., 2024). Much more profound studies and investigations are needed to be applied to give researchers insight into the potential regulatory role and mechanism of circRNAs in PC.

Circ_0001741, a recently discovered circRNA, defined as circTNPO3, which is the location of gene on the chromosome 7: 128655032–12865821 and shares conservative gene region encoding TNPO3 (Li et al., 2022). Recent studies suggest that the aberrant expression of circTNPO3 has been explored in various human malignancies, and it is involved in the regulation of tumor occurrence, progression and even drug resistance. For example, in hepatocellular carcinoma, circTNPO3 regulates STRN expression through competitively sponging miR-199b-5p to promote cell proliferation, migration and invasion, meanwhile, inhibit cell apoptosis (Liu and Liu, 2023); In ovarian cancer, circTNPO3 facilitates paclitaxel resistance via ceRNA mechanism which could be described as absorbing miR-1299 and interfering with the downstream NEK2 (Xia et al., 2020). To date, however, there has not been any research on the biological function of circTNPO3 in PC. In the present study, the function of circTNPO3 in regulating the malignant phenotype of PC were explored. Mechanically, it was suggested that circTNPO3 promoted malignant biological behavior and chemoresistance of PC via miR-188-5p/CDCA3/TRAF2/NF-κB signaling pathway. Meanwhile, the potential of circTNPO3 as a new therapeutic target was also validated through treatment with a novel small molecule antitumor monomer from traditional Chinese medicine. The current research will contribute to elucidate the pathological mechanism of PC and provide new theoretical basis for diagnosis and treatment.

## 2 Materials and methods

### 2.1 Cell culture, chemoresistant cell line establishment and experimental medicine preparation

Human pancreatic cancer cells (including AsPC-1, BxPC-3, CFPAC-1, PANC-1 and SW1990) and a normal human pancreatic duct epithelial cell line (HPDE6-C7) were commercially obtained from National Model and Characteristic Laboratory Cell Resource Bank, Chinese Academy of Sciences (Shanghai, China) and Bnbio Biotechnology Research Institute (Beijing, China). IMDM/DMEM/RPMI-1640 (Gibco, Grand Island, NY, United States) medium containing 10% FBS (C04001-500, VivaCell, Shanghai, China) and 1% penicillin-streptomycin mix solution (SV30010, HyClone, Logan, UT, United States) were applied to culture cells in 37°C conventional incubator with 5% CO_2_. Gemcitabine (GEM) and deoxyelephantopin (DET) were purchased from Gloria Pharmaceuticals (Harbin, China) and BioBioPha (Kunming, China), and dissolved in cell culture grade saline and dimethyl sulfoxide (DMSO) to prepare stock solutions, the specific procedures have been validated in our previous studies (Ji et al., 2020). The DET formula is C_19_H_20_O_6_, molecular weight is 344.36, and CAS number is 29307-03-7. The GEM-resistant PC cell line (BxPC-3/GR and CFPAC-1/GR) used in present study was induced through continuous different concentration of GEM stimulation (Zhou et al., 2020). Finally, the BxPC-3/GR and CFPAC-1/GR cell lines that could persist in the concentration of 1 μM (for BxPC-3/GR) or 500 nM (for CFPAC-1/GR) GEM were obtained. To ensure better cells status, a fresh tube of frozen cells was resuscitated at an interval of 3 months and the *mycoplasma* detection (CA1080, Solarbio, Beijing, China) was conducted.

### 2.2 Patient clinicopathological specimens

A total of 74 pairs of PC tissue samples, matched paracancerous tissue samples and clinicopathological data were jointly acquired from the Second and the Forth Affiliated Hospital of Harbin Medical University. Each tissues were divided into two parts, one part was directly cryopreserved in liquid nitrogen and the other part was immersed into 4% paraformaldehyde solution. The written informed consent was obtained from all patients involved in our present study. Additionally, the operation also obtained approval from the Ethics Board of Harbin Medical University and followed the basic principles of the Declaration of Helsinki.

### 2.3 Cell proliferation assay

Cell Counting Kit-8 (CCK-8) and 5-ethynyl-2' -deoxyuridine (EdU) assays were carried out to evaluate the cell viability. For CCK-8 assay, 3 × 10^3^ cells were inoculated into 96-well plates and cultured for different time points. After that, 10 μL of CCK-8 reagent (GK10001, Glpbio, Montclair, CA, United States) was added to each well and continued to incubate for 2.5 h. Finally, the specific absorbance value at 450 nm was obtained with a multimode microplate analyzer (SCR_024560, Tecan, Mannedorf, Switzerland) and the cell viability was calculated. EdU assay was conducted using EdU-555 Cell Proliferation Kit (C0075S, Beyotime, Shanghai, China). The cells were incubated with 10 μM EdU working solution for 2 h and then fixed with paraformaldehyde solution. After staining the nucleus with Hoechst 33342, the EdU positive labeling cells were analyzed under a fluorescent microscope (SCR_000011, Leica, Wetzlar, Germany).

In order to further examine the ability of cell proliferation, clone forming approaches were performed. 800 cells were seeded into 6-well plated and continuously cultured for 10-12 days under conventional conditions. Next, the visible cell colonies which contained at least 50 cells in each well were fixed and stained. Finally, the colonies were photographed under a microscope and the clone formation rates between different groups were assessed.

### 2.4 Cell migration and invasion assays

The mobility of PC cells was primarily determined using scratch wound healing assay. In brief, cells on logarithmic phase were inoculated into 6-well plate, and a 200 μL sterile micropipette tip and experimental ruler were utilized to scratch longitudinally straight cell-free areas. After rinsing away floating cells with PBS buffer, the cells were cultured under FBS-free medium, and the percentage of regional confluence from different points was compared using inverted microscope.

The migration and invasion abilities of PC cells were further detected using Transwell assays (725321, Nest, Wuxi, China) holding chambers with 8 μm aperture polyethylene terephthalate membrane. PC cells (5 × 10^4^ cells for BxPC-3 and SW1990, 4 × 10^4^ cells for CFPAC-1 and 6 × 10^4^ cells for PANC-1) resuspended in 200 μL FBS-free medium were seeded into the upper chamber module, and 600 μL complete medium were added to the lower chamber module as the putative chemoattractant. After 24 h routine culture, the cells adherent to the lower outer-field portion of the upper chamber were fixed with 4% paraformaldehyde and stained with 0.1% crystal violet aqueous solution. For invasion assay, the membrane inner side of upper chamber module was pre-enveloped with 100 μL Matrigel matrix (HY-K6001, MCE, Princeton, NJ, United States) and the remaining steps were essentially same. The numbers of cell permeating membrane were counted under inverted microscope.

### 2.5 Cell apoptosis assay

Acridine Orange (AO)/Ethidium Bromide (EB) and Hoechst 33342 fluorescent staining assays were applied for apoptosis detection of PC cells. For AO/EB assay, cells were seeded in 48-well culture plate with a density of 1 × 10^4^ per well. After stimulation with different strategies, the cells were rinsed with PBS buffer, then the AO and EB reagents were added at a concentration of 100 μg/mL. After 20 min of reaction, the apoptosis rate was calculated via the ratio of green fluorescence to red fluorescence obtained by a fluorescence microscope (SCR_000011, Leica, Wetzlar, Germany). In Hoechst 33342 assay, the method of cell inoculation and stimulation was the same as above. After fixation with 4% paraformaldehyde for 15 min, 200 μL of Hoechst 33342 (40731ES, Yeasen, Shanghai, China) were dripped into culture well and incubated for another 20 min. The apoptosis was assessed by relatively strong bright blue fluorescence.

Flow cytometry assay was performed to further evaluate the cell apoptosis. In brief, after treatment with different conditions, the cells were collected with 0.25% EDTA-free trypsin. After rinsed three times with PBS and filtered with a 200-mesh strainer, the cells were resuspended in 500 μL buffer solution, then 5 μL of PI and 5 μL Annexin-Ⅴ (556547, BD Biosciences, Franklin Lakes, NJ, United States) were added. The apoptosis ratio was determined through the sum of data from Q2 and Q4 quadrants. Q1 quadrant represents the mechanically damaged cells. Q2 quadrant represents the non-viable apoptotic cells. Q3 quadrant represents the normal viable cells. Q4 quadrant represents the viable apoptotic cells.

### 2.6 Spheroid formation assay

The stem cells self-renewal was evaluated using three-dimensional spheroid formation assay. Briefly, 600 cells were seeded in 24-well ultra-low adhesion culture plate (3,473, Corning, NY, United States) with 500 μL optimal stem cell culture medium per well, and 50 μL medium was added every 2 days. After 2 weeks of routine cell incubation, the number of spheroid with a diameter ≥75 μm was counted under microscope, and the spheroid formation rate was calculated. The stem cell culture medium was composed of DMEM/F12 basic medium appended with 1×B27, 20 ng/mL bFGF, 20 ng/mL EGF and 4 μg/mL insulin.

### 2.7 Bioinformatics analysis and dual-luciferase reporter assay

The target genes of circTNPO3 were predicted through CircBank (http://www.circbank.cn/), ENCORI (https://rnasysu.com/encori/) and TargetScan (https://www.targetscan.org/vert_80/). The binding sites between miR-188-5p and CDCA3 were predicted by ENCORI. The expression level of CDCA3 in PC tissues and its relationship with patient survival were analyzed using TCGA (https://www.cancer.gov/ccg/research/genome-sequencing/tcga), GEPIA (http://gepia.cancer-pku.cn/) and TNMplot (https://tnmplot.com/analysis/) databases. The correlation analysis between CDCA3, TRAF2 and RelA (NF-κB-p65) was carried out based on the TIMER2.0 database (http://timer.cistrome.org/).

In order to explore the binding capacity between circTNPO3 and miR-188-5p, and between miR-188-5p and CDCA3, Dual-Luciferase Reporter Assay (E2920, Promega, Madison, WI, United States) was applied. Briefly, the wild type (WT)/mutant (MUT) of circTNPO3/CDCA3 gene fragments were designed and added to pmirGLO vector. After transfection of blank control vector, mimics negative control and miR-188-5p mimics into cells using Lipofectamine 3000, the fluorescence signal intensity was detected.

### 2.8 Protein extraction and immunoblotting assay

The total protein was extracted from cells under different conditioned stimulus using RIPA lysis buffer added with protease inhibitor (G2006, Servicebio, Wuhan, China). After cell spatula scraping and ultrasonic concussion, the protein lysis was collected using 4°C low temperature precooling centrifuge at 12,000×*g* for 15 min. To detect the translocation of NF-κB-p65 from cytoplasm to nucleus, the Nuclear and Cytoplasmic Protein Extraction Kit (P0027, Beyotime, Shanghai, China) was applied. The protein concentration was quantified using Pierce BCA Protein Assay Kit (23227, Thermo Scientific, Waltham, MA, United States). After denaturation at 100°C metal bath for 5 min, the protein was separated using 10% SDS-PAGE (G2067-50T, Servicebio, Wuhan, China) and transferred to 0.45 μm pore size PVDF blotting membrane (10600029, Cytiva, Wilmington, DE, United States). Then, the PVDF membrane was blocked with 5% (W/V) skim milk for 1 h and incubated with primary antibody overnight at 4°C. Finally, after incubation with the second antibody, the exposure of protein blot was achieved using enhanced chemiluminescence agent (MA0186, Meilunbio, Dalian, China) under biomolecular imaging system (Tanon, Shanghai, China). The primary antibodies involved in this study were listed below: rabbit anti-CDCA3 antibody (bs-7894R, 1:1,500, Bioss, Beijing, China), rabbit anti-TNPO3 antibody (T58524S, 1:2000, Abmart, Shanghai, China), rabbit anti-GRPDH antibody (ab181602, 1:8,000, Abcam, Shanghai, China), rabbit anti-cleaved-caspase 9 antibody (AF5240, 1:1,500, Affinity, Jiangsu, China), rabbit anti-cleaved-caspase 3 antibody (AF7022, 1:1,500 Affinity, Jiangsu, China), rabbit anti-TRAF2 antibody (T55841S, 1:2000, Abmart, Shanghai, China), mouse anti-p65 antibody (sc-8008, 1:1,000, Santa Cruz, Dallas, TX, United States).

### 2.9 RNA isolation, reverse transcription and quantitative real-time PCR (qRT-PCR)

After conditioned stimulus, the total RNA, cytoplasmic and nuclear RNA were extracted using RNAsimple Total RNA Kit (DP419, TIANGEN, Beijing, China) and Cytoplasmic and Nuclear RNA Purification Kit (NGB-21000, NORGEN, Ontario, Canada), respectively. The cDNA synthesis and real-time PCR were conducted using QuantiTect Reverse Transcriptase Kit (205311, QIAGEN, Redwood City, CA, United States) and Talent qPCR PreMix (FP209, TIANGEN, Beijing, China) according to the supplier’s protocol. U6 and GAPDH were selected as a reference gene, the quantitative expression of target genes was analyzed through 2^−ΔΔCt^ formula. The primer sequences for qRT-PCR assay were listed in Supplementary Table S1.

### 2.10 RNA stability and half-life detection

The circular attribute of circTNPO3 was confirmed using RNase R (LGCRNR07250, Sigma, Taufkirchen, Germany) treatment. Total RNA was extracted from PC cells and the concentration was detected by applying ultraviolet spectrophotometer. Next, a certain amount of total RNA (≤5 μg) was treated with RNase R in a ratio of 3 U/μg RNA. After co-incubation at 37°C for 20 min, the RNA was isolated and purified by the TRIzol Plus RNA Purification Kit (12183555, Thermo Scientific, Waltham, MA, United States).

The half-life of target mRNA and circRNA was monitored by administering actinomycin D (A4262, Sigma, Taufkirchen, Germany). After cells were cultured in medium containing10 μg/mL of actinomycin D for different duration, the total RNA was extracted. Finally, the half-life of RNA was analyze through reverse transcription and qRT-PCR.

### 2.11 Fluorescence *in situ* hybridization (FISH)

The subcellular localization of circTNPO3 in PC cells was detected using Fluorescence *in Situ* Hybridization Kit for RNA (R0306S, Beyotime, Shanghai, China) according to the supplier’s instructions. PC cells were seed in 35 mm confocal petri dishes, fixed with 4% polyformaldehyde and permeated with proteinase K. Then the cells were co-incubated with Cy3-labeled circTNPO3 antisense probes (designed and synthesized by GenePharma, Suzhou, China) in hybridization solution overnight. After nucleus counterstained with DAPI, circTNPO3 localization was analyzed through the distribution of red fluorescence.

### 2.12 *In situ* hybridization (ISH)

The expression of circTNPO3 in PC specimens was tested by digoxin (DIG)-labeled circTNPO3 probes (synthesized by GenePharma, Suzhou, China). Paraffin sections of PC tissues were deparaffinized by different concentrations of xylene and ethanol, executed antigen retrieval using proteinase K and co-incubated with circTNPO3 probes at 40°C for 12 h. The signals of circTNPO3 and nucleus in pathologic tissue slices were displayed after visualization processing by applying anti-DIG-POD antibody, DAB chromogenic reagent and hematoxylin, respectively. The expression level of circTNPO3 was quantified through staining intensity (scored: 0, none; 1, light brown; 2, brown; 3, dark brown) and percentage of positively stained cells (scored: 1, <25%; 2, 25%-50%; 3, 50%-75%, 4, 75%-100%) comprehensively.

### 2.13 *In vivo* oncogenicity

Female BALB/c nude mice aged 4–6 weeks were purchased from Charles River Laboratories (Beijing, China) and raised in individual ventilated cages system (IVC) with the standard of specific pathogen free (SPF). After acclimation for 1 week, the lab mice were randomly divided into 3 groups of 5. In the subcutaneously xenograft tumor model, 100 μL of cell suspension containing 4 × 10^6^ conditionally stimulated cells were subcutaneously grafted into the left axilla of nude mice. Tumor growth curves were plotted using the tumor volume measured every 3 days. The tumor volume was calculated via the following formula: Tumor volume = π/6 × longest diameter × shortest diameter^2^. After 36 days of aborative feeding, the mice were sacrificed through spinal cord transaction. The tumors were dissected, weighed, measured and split in two parts, with one soaked in 4% paraformaldehyde solution and the other preserved in liquid nitrogen immediately. To investigate the effect of circTNPO3 on metastasis of PC, the lung metastatic tumor model was constructed. 200 μL of cell suspension containing 3 × 10^6^ cells were transferred into mice lung via rapid tail vein injection using Vascular Visualization System (Yiyan, Jinan, China). After 6 weeks of meticulous care, the mice were euthanized. And the lungs were dissected and preserved in fixative solution for hematoxylin-eosin staining. The level of lung metastasis was comprehensively assessed through the number of globular nodules and the lesion of pulmonary alveoli. The above animal research has been approved by the Ethics Committee of Harbin Medical University, and the review file number is SYDW2020-058.

### 2.14 Statistical analysis

SPSS Statistics 28.0.1 software (https://www.ibm.com/docs/zh/spss-statistics) was used for quantitative analysis of data. The experimental data in the present research were repeated at least three times, and listed as the mean ± standard deviation (SD). The statistical diagrams were visualized using GraphPad Prism 8 (https://www.graphpad-prism.cn/). The Student’s *t*-test was introduced to analyze the difference between the two sets of data. Kaplan–Meier curve was applied to compare the overall survival of cohorts with different circTNPO3 expression levels. The correlations between miR-188-5p, CDCA3, TRAF2 and NF-κB-p65 were analyzed using Pearson correlation coefficient, respectively. *P* value of less than 0.05 was defined as statistically significant and marked with asterisk (*) or pound sign (#).

## 3 Results

### 3.1 CircTNPO3 expression is upregulated in PC and associated with poor prognosis

**FIGURE 1:**
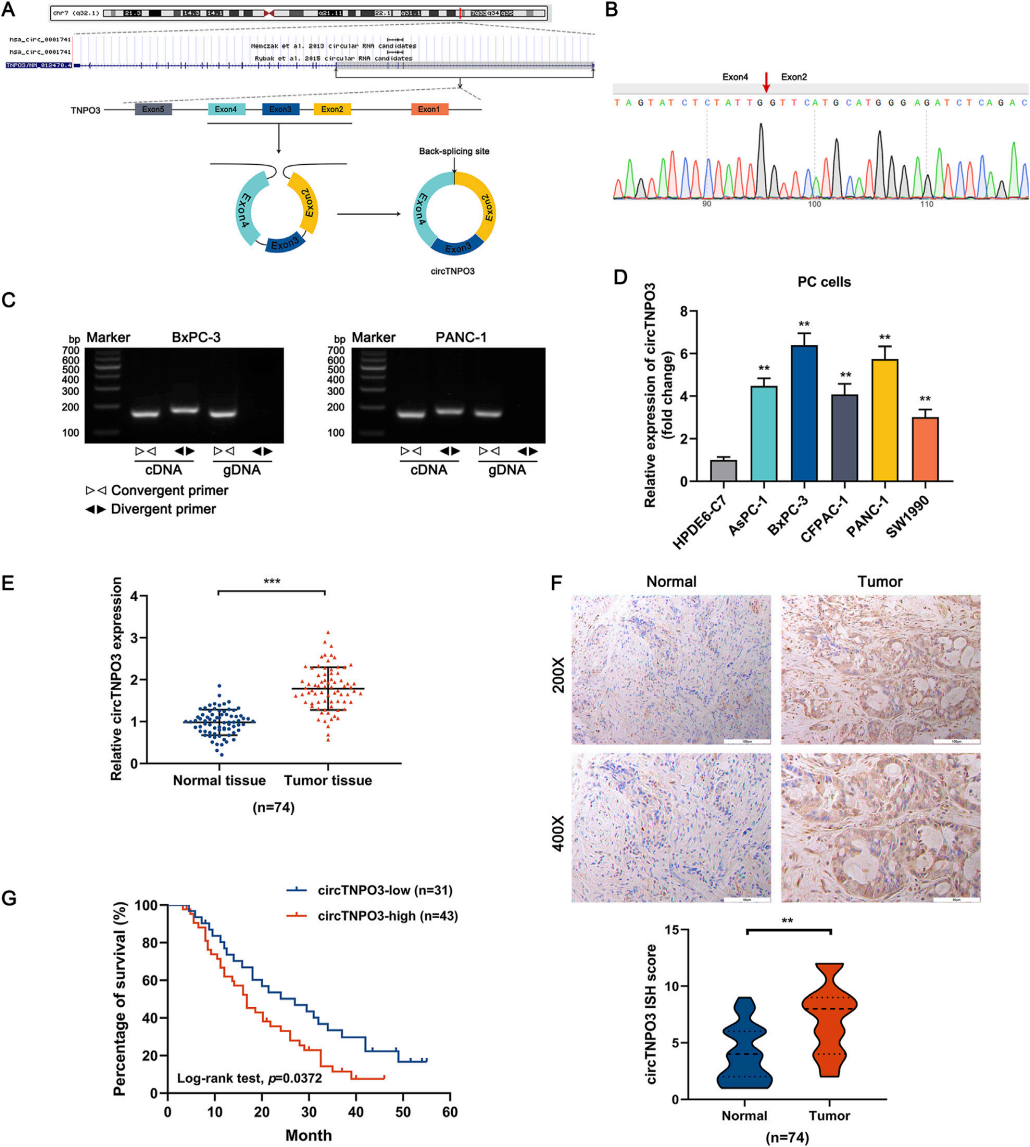
The heterogeneity of circTNPO3 in PC cells and tissues, and association with clinicopathological characteristics. **(A)** The chromosome localization and splicing schematic of has_circ_0001741 (circTNPO3) were presented. **(B)** The specific primers designed for circTNPO3 were validated by qRT-PCR and the PCR product was verified through Sanger sequencing. The red arrow represented the back-splicing site. **(C)** The cyclization characteristic of circTNPO3 was confirmed through agarose gel electrophoresis using divergent and convergent primers in BxPC-3 and PANC-1 cells. **(D)** The differential expression of circTNPO3 between human normal pancreatic ductal epithelial (HPDE6-C7) and PC (AsPC-1, BxPC-3, CFPAC-1, PANC-1 and SW1990) cell lines was detected by qRT-PCR. **(E)** qRT-PCR analysis of circTNPO3 expression between 74 pairs of PC tissues and adjacent peritumoral tissues. **(F)** The expression of circTNPO3 in PC tissues and adjacent peritumoral tissues was detected by ISH assay. (*n* = 74). **(G)** The overall survival of PC patients with lower (*n* = 31) and higher (*n* = 43) circTNPO3 expression was compared using Kaplan–Meier curve. ∗∗*P* < 0.01, ∗∗∗*P* < 0.001. Magnification, ×200 (first row of F), × 400 (second row of F). Scale bar, 100 μm (first row of F), 50 μm (second row of F). PC, pancreatic cancer. ISH, *in situ* hybridization.

Based on the chromosome localization and splicing schematic (Figure 1A) of has_circ_0001741 (circTNPO3), the divergent primers crossing the junction (cyclization of exons 2-4 of a TNPO3 mRNA transcript) were designed. As shown in Figure 1B, the Sanger sequencing verified the back-splicing site of circTNPO3. In order to illustrate the cyclization characteristic of circTNPO3, the primers for amplifying circular and linear TNPO3 were synthesized. As shown in Figure 1C, the data of agarose gel electrophoresis implied that circTNPO3 could only be amplified from cDNA of BxPC-3 and PANC-1 cells using divergent primers, not from gDNA. By comparison, the linear TNPO3 could be amplified from both cDNA and gDNA using convergent primers. Next, the expression level of circTNPO3 in PC cell lines was analyzed using qRT-PCR. As shown in Figure 1D, the expression of circTNPO3 was significantly increased in PC cells (AsPC-1, BxPC-3, CFPAC-1, PANC-1 and SW1990) compared with that in normal human pancreatic ductal epithelial (HPDE6-C7) cells. Similar status were verified in a cohort of PC tissues and corresponding paracancer tissues collected from 74 patients, indicating that the expression of circTNPO3 was higher in tumor tissues than that in the normal tissues (Figure 1E). This differential trend was further validated in ISH assay (Figure 1F). Next, the 74 patients were classified into lower-expression (*n* = 31) and higher-expression (*n* = 43) subgroups through setting the median expression of circTNPO3 as a criterion. The Kaplan-Meier survival curve analysis suggested that patients with lower circTNPO3 expression obtained better overall survival times than those with higher circTNPO3 expression (Figure 1G).

### 3.2 CircTNPO3 modulates the proliferation, migration, invasion and apoptosis characteristics of PC cells

**FIGURE 2:**
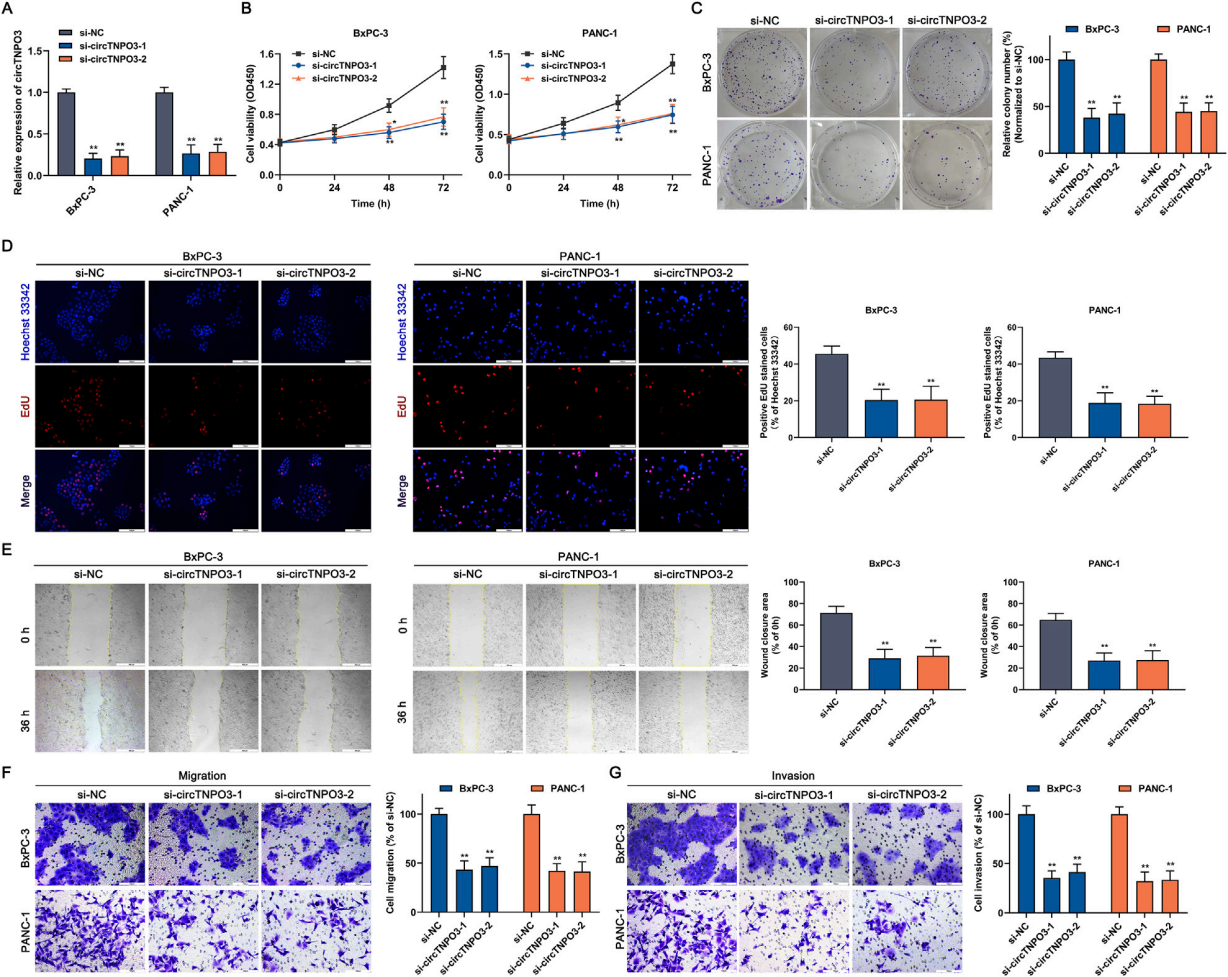
Silencing circTNPO3 suppressed the proliferation, migration and invasion of PC cells *in vitro*. **(A)** The expression of circTNPO3 in BxPC-3 and PANC-1 cells after transfection with si-NC or interference sequences targeting circTNPO3 (si-circTNPO3-1/2) was detected by qRT-PCR. **(B)** The viability of BxPC-3 and PANC-1 cells after transfection with siRNAs was assessed by CCK-8 assay. **(C)** The proliferation of BxPC-3 and PANC-1 cells was analyzed by colony forming assay. **(D)** The EdU positive staining representing proliferation of BxPC-3 and PANC-1 cells was quantified by EdU assay. **(E)** Wound healing assay was performed to detect the locomotive ability of BxPC-3 and PANC-1 cells after circTNPO3 silencing. **(F,G)** Transwell assay was conducted to assess the migration and invasion of BxPC-3 and PANC-1 cells. *P < 0.05, **P < 0.01. Magnification, ×40 **(E)**, × 200 **(D,F,G)**. Scale bar, 500 μm **(E)**, 100 μm **(D,F,G)**. EdU, 5-Ethynyl-2′-deoxyuridine.

**FIGURE 3:**
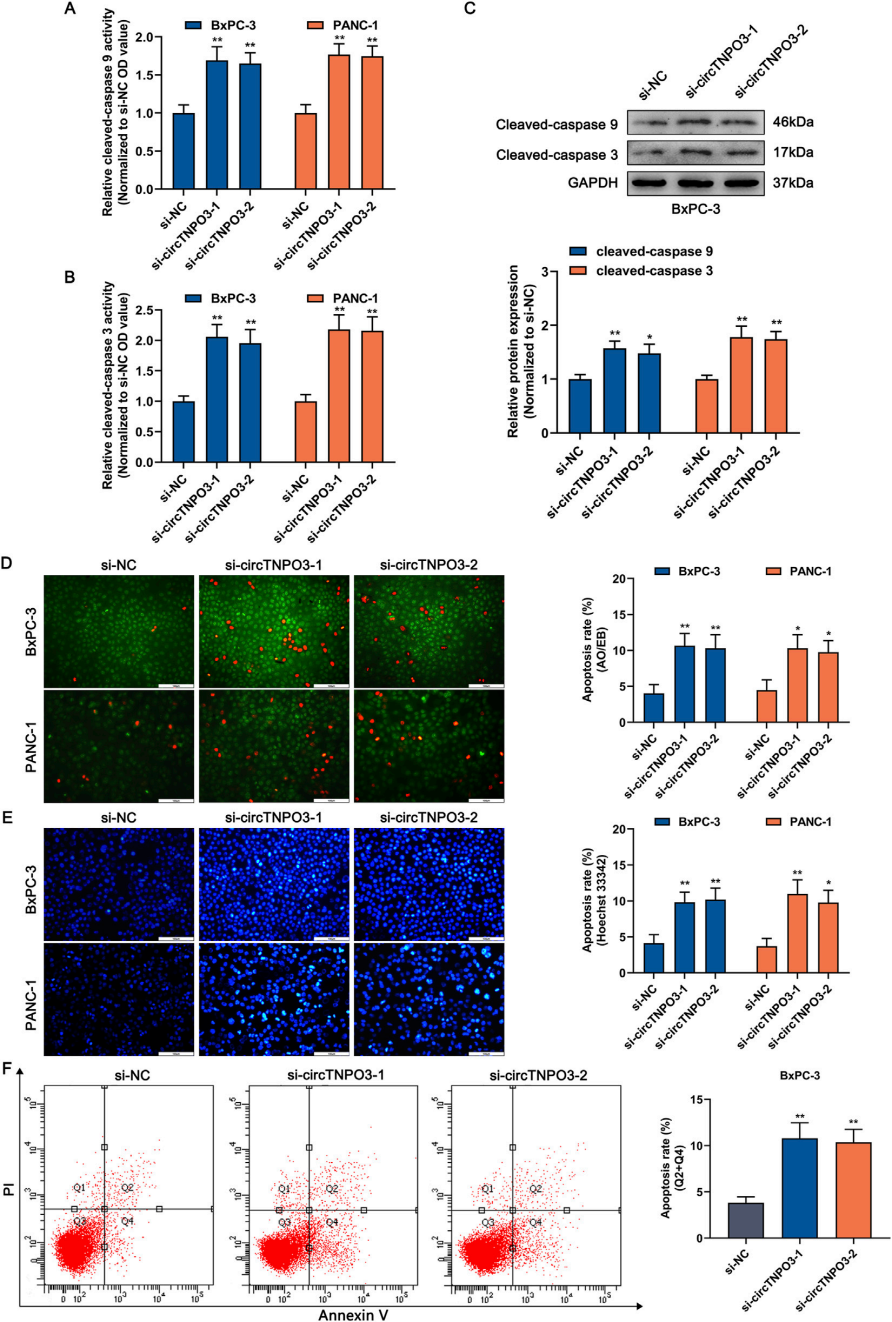
Silencing circTNPO3 induced the apoptosis of PC cells. **(A,B)** The activation of apoptosis-associated markers including upstream caspase 9 and downstream caspase 3 was determined using Caspase 9 and Caspase 3 Activity Assay Kits. **(C)** The expression level of cleaved-caspase 9 and cleaved-caspase 3 after circTNPO3 silencing was measured using immunoblotting in BxPC-3 cells. **(D,E)** The apoptosis rate of BxPC-3 and CFPAC-1 cells after circTNPO3 silencing was detected by AO/EB and Hoechst 33342 fluorescent staining assays. **(F)** Flow cytometry analysis was applied to analyze the apoptosis of BxPC-3 and CFPAC-1 cells after circTNPO3 silencing. Q1 quadrant, mechanically damaged cells. Q2 quadrant, non-viable apoptotic cells. Q3 quadrant, normal viable cells. Q4 quadrant, viable apoptotic cells. *P < 0.05, **P < 0.01. Magnification, ×200 **(D,E)**. Scale bar, 100 μm **(D,E)**. AO, Acridine Orange. EB, Ethidium Bromide.

To explore the role of circTNPO3 in PC cells, function loss and gain strategies were carried out by transfecting circTNPO3-siRNAs, circTNPO3-shRNAs or circTNPO3-overexpresion lentiviruses. Firstly, circTNPO3 was downregulated by si-circTNPO3-1/2 in BxPC-3 and PANC-1 cells with comparatively higher expression level. The knockdown efficiency was confirmed using qRT-PCR, suggesting that the expression of circTNPO3 was indeed downregulated in si-circTNPO3-1/2 group compared with that in si-NC group (Figure 2A). Moreover, the unique targeting property of si-circTNPO-1/2 was confirmed by immunoblotting assay, showing that the expression of linear gene TNPO3 was not intervened (Supplementary Figure S1A). The CCK-8 and colony formation assays showed that the cell viability and proliferation of BxPC-3 and PANC-1 were significantly inhibited in si-circTNPO3-1/2 group (Figures 2B, C). The EdU assay exhibited the similar results (Figure 2D). However, although the circTNPO3 was efficiently knockdown in HPDE6-C7 cells, significant changes in cell viability were not found (Supplementary Figures S1B,C). This result indicated that circTNPO3 might act as a tumor specific gene in PC. The wound healing and Transwell assays found that the migration and invasion abilities of PC cells were suppressed in circTNPO3 silencing group compared with that in control group (Figures 2E–G). Furthermore, the tendency of apoptotic cells was assessed through canonical apoptosis-associated makers. As shown in Figures 3A,B, the silence of circTNPO3 promoted the conversion of pro-apoptotic protein caspase 9 and downstream caspase 3 to activated form. Meanwhile, the elevation in the expression of cleaved-caspase 9 and cleaved-caspase 3 was detected by immunoblotting assay (Figure 3C). The AO/EB, Hoechst 33342 and flow cytometry assays also showed that the apoptosis rate was higher in si-circTNPO3-1/2 groups, implying that the effect of circTNPO3 on the proliferation of PC cells might be partly dependent on the regulation of apoptosis (Figures 3D–F).

**FIGURE 4:**
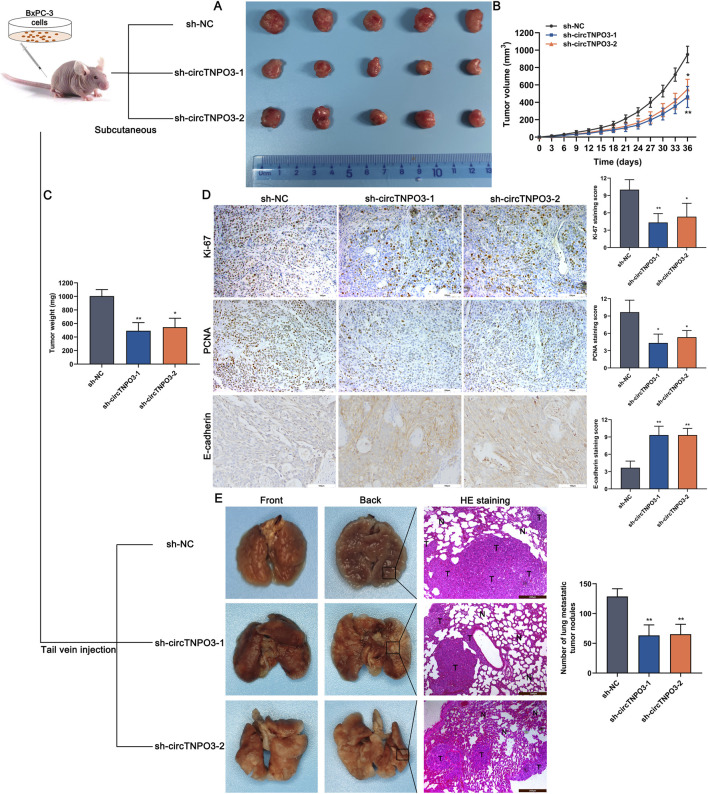
Silencing circTNPO3 attenuated PC growth and metastasis *in vivo*. **(A)** BxPC-3 cells after transfection with sh-NC or sh-circTNPO3-1/2 were inoculated into the left axilla of nude mice to construct subcutaneous xenograft model, and the mice were divided to three groups. (*n* = 5). **(B)** Tumor growth curves were plotted according to the tumor volume recorded every 3 days. **(C)** The final weight of the subcutaneous tumor. **(D)** The functional protein associated with proliferation (Ki-67, PCNA) and EMT (E-cadherin) in tumor samples were detected by IHC assay. **(E)** The lung metastatic model was established by nude mice tail vein injection of transfected BxPC-3 cells, and the alveolar structure was evaluated by H&E staining. (*n* = 5). *P < 0.05, **P < 0.01. Magnification, ×100 **(E)**, × 200 **(D)**. Scale bar, 200 μm **(E)**, 100 μm **(D)**. H&E, hematoxylin-eosin. EMT, epithelial to mesenchymal transition.

*In vivo* experiments were conducted to estimate the effect of circTNPO3 on PC growth and metastasis. The data from subcutaneous graft tumor model suggested that the tumor growth velocity and final volume were significantly reduced in sh-circTNPO3-1/2 groups as compared with that in the sh-NC group (Figures 4A–C). And the above results were supported by the decreased expression of proliferation-related proteins, such as Ki-67 and PCNA (Figure 4D). Additionally, the lower number of lung metastasis nodules, more complete alveolar structure and increased expression of EMT-related marker E-cadherin showed that silence of circTNPO3 also attenuated the metastatic ability of PC (Figure 4E).

**FIGURE 5:**
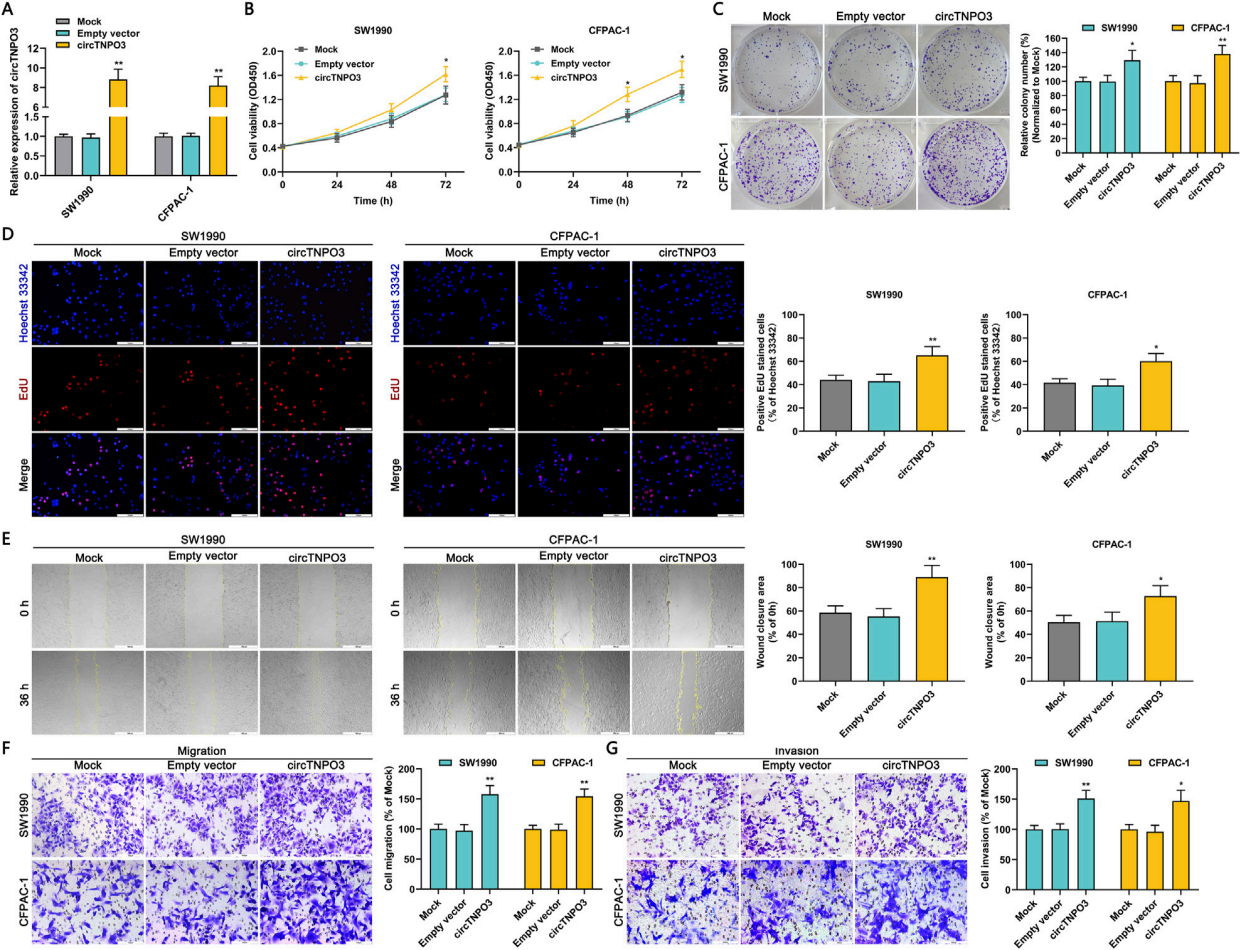
Overexpression circTNPO3 promoted the proliferation, migration and invasion of PC cells *in vitro*. **(A)** The expression of circTNPO3 in SW1990 and CFPAC-1 cells after transfection with empty vector or overexpression vector was detected by qRT-PCR. **(B)** The viability of SW1990 and CFPAC-1 cells after circTNPO3 overexpression was analyzed by CCK-8 assay. **(C)** The proliferation of SW1990 and CFPAC-1 cells after circTNPO3 overexpression was assessed by colony forming assay. **(D)** The EdU positive staining representing proliferation of SW1990 and CFPAC-1 cells after circTNPO3 overexpression was quantified by EdU assay. **(E)** The locomotive ability of SW1990 and CFPAC-1 cells after circTNPO3 overexpression was tested by wound healing assay. **(F,G)** The migration and invasion of SW1990 and CFPAC-1 cells after circTNPO3 overexpression was analyzed by Transwell assay. *P < 0.05, **P < 0.01. Magnification, ×40 **(E)**, × 200 **(D,F,G)**. Scale bar, 500 μm **(E)**, 100 μm **(D,F,G)**.

In order to further investigate the regulatory role of circTNPO3, the circTNPO3 was overexpressed in SW1990 and CFPAC-1 cells with comparatively lower expression level. *In vitro* experiments data showed that circTNPO3 overexpression promoted PC cells proliferation, migration and invasion (Figure 5). The phased research data confirmed that circTNPO3 might act as an oncogene in PC.

### 3.3 CircTNPO3 is highly expressed in GEM chemoresistance cells and decreases chemosensitivity of PC *in vitro*

**FIGURE 6:**
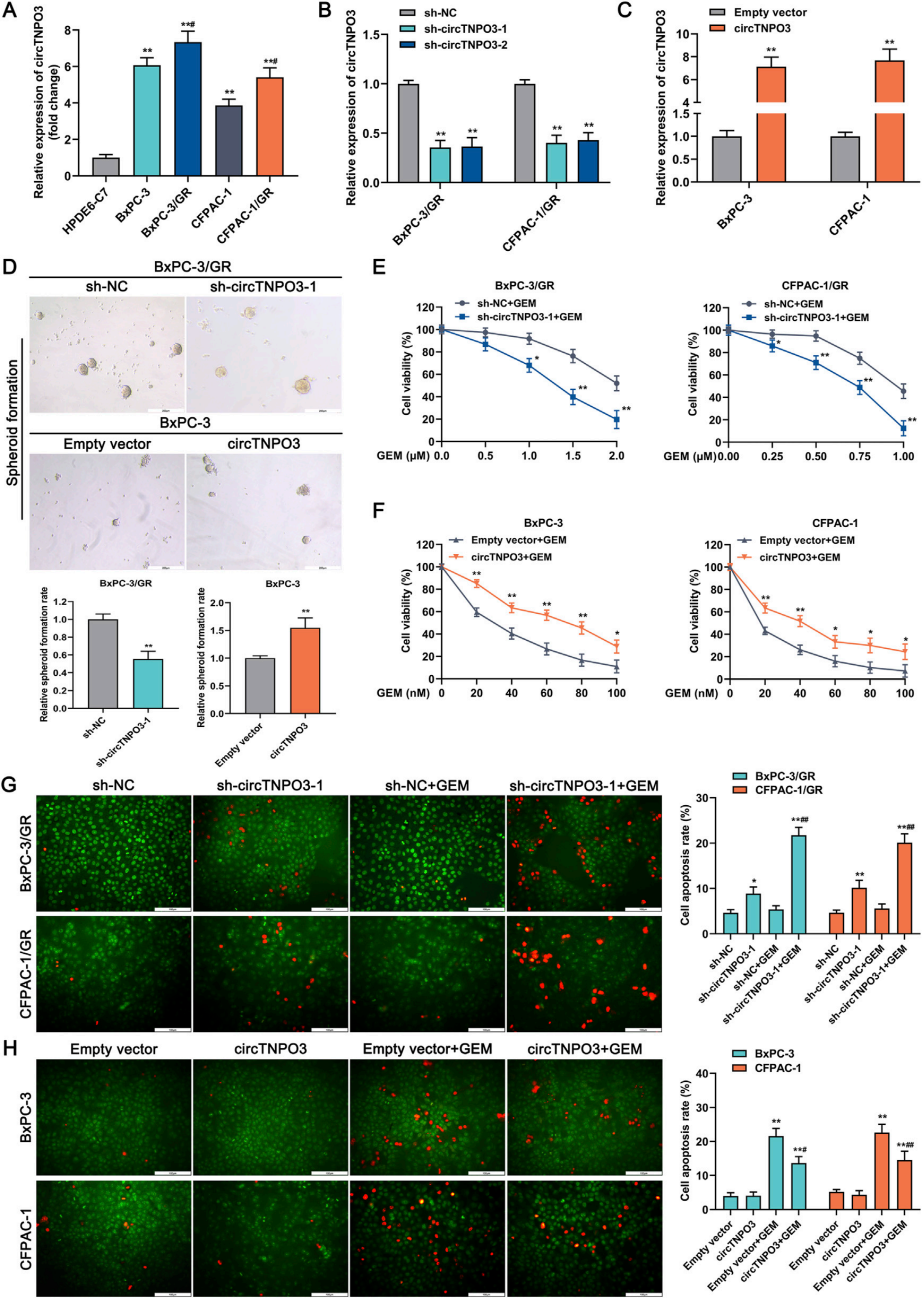
CircTNPO3 was involved in the regulation of GEM chemoresistance in PC. **(A)** The differential expression of circTNPO3 between chemoresistant PC cells and non-chemoresistant parental PC cells was analyzed using qRT-PCR. **P < 0.01 versus HPDE6-C7 group. #*P* < 0.05 versus respective parental non-chemoresistant control (BxPC-3, CFPAC-1) group. **(B)** The expression of circTNPO3 after transfection with sh-circTNPO3-1/-2 was detected using qRT-PCR. **(C)** The expression of circTNPO3 after transfection with overexpression vector was monitored by qRT-PCR. **(D)** Stem cell self-renewal associated with chemoresistance was tested using spheroid formation assay. **(E)** The viability of BxPC-3/GR and CFPAC-1/GR cells after circTNPO3 silencing combined with GEM treatment was tested by CCK-8 assay. **(F)** The viability of BxPC-3 and CFPAC-1 cells after circTNPO3 overexpression combined with GEM treatment was tested by CCK-8 assay. *P < 0.05, **P < 0.01. Magnification, ×100 **(D)**. Scale bar, 200 μm **(D)**. **(G)** The apoptosis rate of BxPC-3/GR and CFPAC-1/GR cells after circTNPO3 silencing combined with GEM treatment was detected by AO/EB fluorescent staining assay. **(H)** The apoptosis rate of BxPC-3 and CFPAC-1 cells after circTNPO3 overexpression combined with GEM treatment was detected by AO/EB fluorescent staining assay. *P < 0.05, **P < 0.01 versus sh-NC group. #*P* < 0.05, ##*P* < 0.01 versus sh-NC combined GEM or empty vector combined GEM group. GEM, gemcitabine. BxPC-3/GR, BxPC-3 gemcitabine resistant cell line. CFPAC-1/GR, CFPAC-1 gemcitabine resistant cell line.

To explore the effect of circTNPO3 on GEM chemosensitivity of PC, the GEM resistant cell lines BxPC-3/GR and CFPAC-1/GR were established. As shown in Figure 6A, the expression of circTNPO3 was further analyzed in chemoresistant PC cells compared with that in the non-chemoresistant parental PC cells. And, the similar function loss or gain experiments were carried out in GEM resistant cells and non-resistant parental cells (Figures 6B,C). The recent oncology perspectives indicated that the immortality and chemoresistance of tumor cells might be closely associated with cancer stem cells (Sarabia-Sánchez et al., 2024). So the spheroid formation assay which assessed the stem cells self-renewal was performed. As shown in Figure 6D, the spheroid formation rate of GEM resistant cells was suppressed in circTNPO3 silencing group compared with that in the sh-NC group. On the contrary, the spheroid formation rate of non-resistant cells was significantly increased in circTNPO3 overexpression group. Furthermore, the silence of circTNPO3 partially improved GEM chemosensitivity in BxPC-3/GR and CFPAC-1/GR cells, mainly consisting of the diminished cell viability and soaring apoptosis rate induced by GEM cytotoxicity (Figures 6E, G). However, circTNPO3 overexpression stimulated chemoresistance characteristics in parental non-resistant BxPC-3 and CFPAC-1 cells (Figures 6F, H). The available data from our study supported the concept that circTNPO3 not only acted as an oncogene but also a GEM resistance-related factor in PC.

### 3.4 CircTNPO3 participates in the regulation of PC progression and GEM chemoresistance through secluding miR-188-5p that regulates CDCA3

**FIGURE 7:**
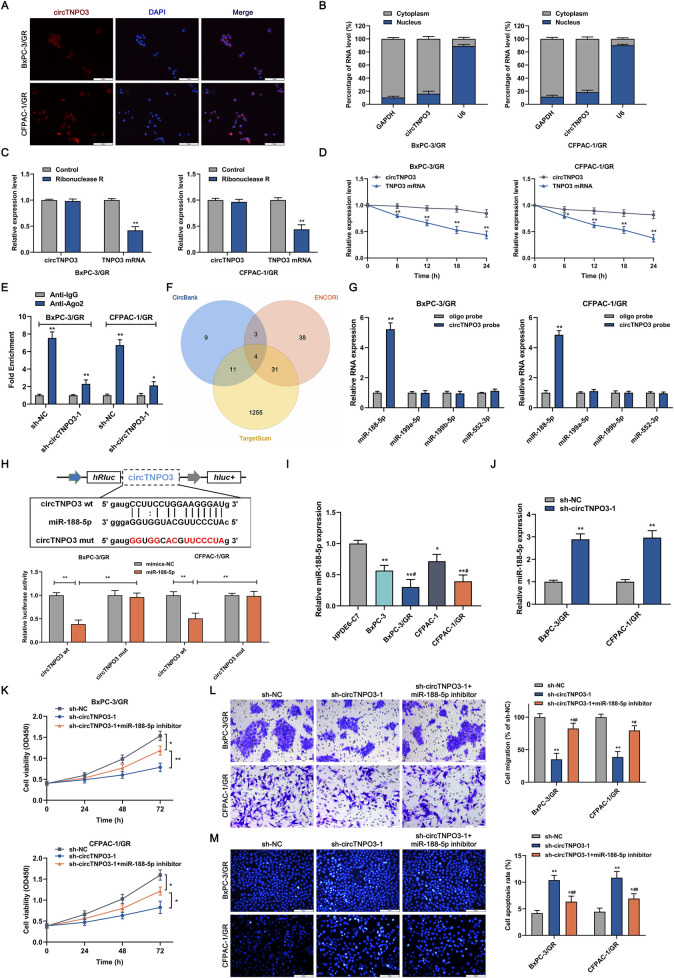
The subcellular localization of circTNPO3 and the identification of interacting miRNAs. **(A)** The localization of circTNPO3 in BxPC-3/GR and CFPAC-1/GR cells was visualized using FISH assay. **(B)** The cellular distribution of circTNPO3 in BxPC-3/GR and CFPAC-1/GR cells was analyzed using Cytoplasmic and Nuclear RNA Purification Kit and qRT-PCR assay. **(C)** The expression of circTNPO3 and corresponding linear TNPO3 mRNA after RNase R treatment in BxPC-3/GR and CFPAC-1/GR cells was tested using qRT-PCR. **(D)** The half-life of circTNPO3 and linear TNPO3 mRNA after actinomycin D treatment in BxPC-3/GR and CFPAC-1/GR cells was assessed using qRT-PCR. **(E)** The binding capacity between circTNPO3 and miRNAs was investigated using Ago2-RIP assay. **(F)** The intersection of the target miRNAs of circTNPO3 was intersectively identified using CircBank, ENCORI and TargetScan databases, and displayed through Venn diagram. **(G)** The binding ability between circTNPO3 and four predicted miRNAs including miR-188-5p, miR-199a-5p, miR-199b-5p and miR-552-3p was verified using RNA pulldown assay. **(H)** The binding sequence between circTNPO3 and miR-188-5p was showed, and the direct binding potential was determined using dual-luciferase reporter assay. *P < 0.05, **P < 0.01. **(I)** The differential expression of miR-188-5p between chemoresistant PC cells and non-chemoresistant parental PC cells was analyzed using qRT-PCR. *P < 0.05, **P < 0.01 versus HPDE-C7 group. #*P* < 0.05 versus respective parental non-chemoresistant control (BxPC-3, CFPAC-1) group. **(J)** The expression of miR-188-5p after circTNPO3 silencing in BxPC-3/GR and CFPAC-1/GR cells was detected using qRT-PCR. **(K)** The viability of BxPC-3/GR and CFPAC-1/GR cells after transfection with sh-circTNPO3-1 and miR-188-5p inhibitor was measured using CCK-8 assay. **(L)** The migration ability of BxPC-3/GR and CFPAC-1/GR cells after transfection with sh-circTNPO3-1 and miR-188-5p inhibitor was determined using Transwell assay. **(M)** The apoptosis of BxPC-3/GR and CFPAC-1/GR cells after transfection with sh-circTNPO3-1 and miR-188-5p inhibitor was evaluated using Hoechest 33342 fluorescence staining assay. *P < 0.05, **P < 0.01 versus sh-NC group. #*P* < 0.05, ##*P* < 0.01 versus sh-circTNPO3-1 group.

Studies have suggested that the regulatory mechanism of circRNA is associated with unique subcellular localization (Zhao et al., 2022). As shown in RNA fluorescence *in situ* hybridization assays based on specific probes and analysis of RNA nucleoplasmic distribution (Figures 7A,B), circTNPO3 was mainly pinpointed in the cytoplasm, implying that circTNPO3 might be involved in gene expression control at the post-transcriptional level. The stability of circTNPO3 was also analyzed to further verify its unique ring configuration. The total RNAs from BxPC-3/GR and CFPAC-1/GR were extracted after treated with RNase R, an exoribonuclease that could induce the RNA degradation from 3′initial to 5′terminal, but not cleave circRNA. As shown in Figure 7C, circTNPO3 exhibited significant resistance to RNase R compared with linear TNPO3 mRNA. The similar data was obtained after the cells treated with actinomycin D, a recognized transcription inhibitor, implying that circTNPO3 held a longer half-time than linear TNPO3 mRNA (Figure 7D). To explore the potential of circTNPO3 to absorb miRNAs through ceRNA mechanism, the Ago2-RIP assay was carried out. As shown in Figure 7E, compared with the blank control group with immunoglobulin-G (IgG) antibody, the enrichment of circTNPO3 was significantly higher in the Ago2 immunoprecipitation group, but was interrupted by circTNPO3 silencing. After comprehensive analysis of bioinformatics databases, including circBank, ENCORI and TargetScan, four target miRNAs, including miR-188-5p, miR-199a-5p, miR-199b-5p and miR-552-3p that might bind to circTNPO3, were predicted (Figure 7F). The qRT-PCR combined RNA pulldown assay based on biotin-labeled circTNPO3 probes showed that compared to the other three miRNAs, only miR-188-5p could be simultaneously enriched in both BxPC-3/GR and CFPAC-1/GR cells (Figure 7G). To determine the direct interaction between circTNPO3 and miR-188-5p, the dual-luciferase reporter assay instructed with wt (wild type) and mut (mutant type) circTNPO3 sequences was performed. Experiment data in Figure 7H found that compared with mimic negative control, the fluorescence signal intensity was decreased by miR-188-5p mimics in circTNPO3-wt group, but no significant difference was observed in circTNPO3-mut reporter gene plasmid. Combined with the expression of circTNPO3 in PC cell lines, the expression of miR-188-5p was shown to be negatively related with circTNPO3, and with the emergence of chemoresistance, its expression level was further reduced (Figure 7I). Conversely, silencing circTNPO3 could significantly upregulate miR-188-5p expression in BxPC-3/GR and CFPAC-1/GR cells, indicating that circTNPO3 might regulate the function of miR-188-5p by direct combination (Figure 7J). Moreover, transfection with miR-188-5p inhibitor partially reversed the inhibition of proliferation and migration, and alleviated the apoptosis caused by circTNPO3 silencing in BxPC-3/GR and CFPAC-1/GR cells (Figures 7K–M).

**FIGURE 8:**
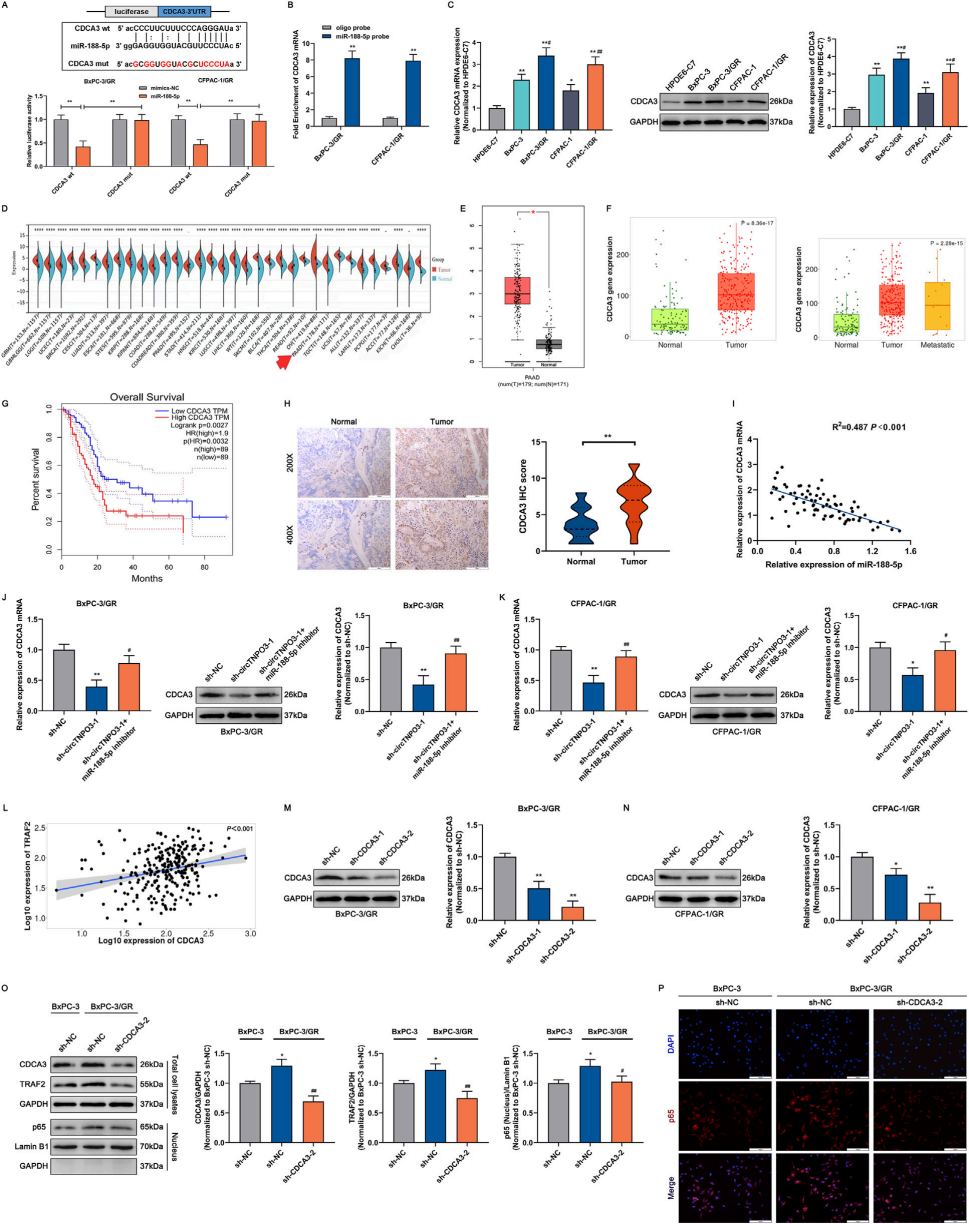
circTNPO3 facilitated GEM chemoresistance through competitively binding miR-188-5p, upregulating CDCA3 and activing NF-κB nuclear translocation. **(A)** The binding sequence between miR-188-5p and CDCA3 mRNA was listed, and the direct binding potential was confirmed using dual-luciferase reporter assay. **(B)** The binding ability between miR-188-5p and CDCA3 mRNA was determined using miR-188-5p probe RNA pulldown assay. **(C)** The differential expression of CDCA3 between chemoresistant PC cells and parental non-chemoresistant PC cells was analyzed using qRT-PCR and immunoblotting assays. *P < 0.05, **P < 0.01 versus HPDE6-C7 group. #*P* < 0.05, ##*P* < 0.01 versus respective parental non-chemoresistant control (BxPC-3, CFPAC-1) group. **(D)** Pan-cancer atlas of CDCA3 in various human malignant tumors, and the differential expression of CDCA3 in PC was labeled by a red arrow. **(E,F)** The expression level of CDCA3 in PC was assessed by TCGA, GTEx and TNMplot databases. **(G)** Kaplan-Meier curve according to GEPIA database was used to analyze the overall survival of PC patients with lower and higher CDCA3 expression level. **(H)** The expression level of CDCA3 between PC tumor tissues and adjacent normal tissues was compared using IHC assay. **(I)** The correlation between miR-188-5p and CDCA3 in PC was analyzed through Pearson correlation coefficient. **(J,K)** The expression level of CDCA3 in BxPC-3/GR and CFPAC-1/GR cells after transfection with sh-circTNPO3-1 and miR-188-5p inhibitor was detected using qRT-PCR and immunoblotting assays. *P < 0.05, **P < 0.01 versus sh-NC group. #*P* < 0.05, ##*P* < 0.01 versus sh-circTNPO3-1 group. **(L)** The correlation between CDCA3 and TRAF2 in PC was analyzed through Pearson correlation coefficient from TNMplot database. **(M,N)** The expression level of CDCA3 in BxPC-3/GR and CFPAC-1/GR cells after transfection with sh-CDCA3-1/-2 was examined using immunoblotting assay. **(O)** The expression level of TRAF2 and the NF-κB-p65 nuclear translocation in BxPC-3 and BxPC-3/GR cells after CDCA3 knockdown were determined using immunoblotting assay. *P < 0.05 versus sh-NC group. ##*P* < 0.01 versus sh-CDCA3-2 group. **(P)** The NF-κB-p65 nuclear translocation in BxPC-3 and BxPC-3/GR cells after CDCA3 knockdown was evaluated using IF assay. IF, immunofluorescence.

In order to unveil the mechanism of circTNPO3 regulating PC malignant phenotype through miR-188-5p, the TargetScan bioinformatics database for predicting downstream target was applied. As shown in Figure 8A, CDCA3 might be the potential downstream target gene of miR-188-5p. Moreover, to detect the binding potential between miR-188-5p and CDCA3, cells were co-transfected with the miR-188-5p mimics designed with the reporter vector containing the 3′-UTR of CDCA3. The results indicated that compared with mimic negative group, the luciferase activity of the reporter vector containing the wt 3′-UTR of CDCA3 was significantly suppressed by miR-188-5p mimics. On the contrary, fluorescence inhibition induced by miR-188-5p was blocked in mutant 3′-UTR of CDCA3 group. This result was verified by RNA pull down assay, compared with the blank control group with oligo probe, the enrichment of CDCA3 mRNA was obviously higher in the experimental group with miR-188-5p probe (Figure 8B). Immunoblotting and qRT-PCR assay found that CDCA3 mRNA and CDCA3 protein were overexpressed in PC cell lines compared with that in HPDE6-C7 cell line, and were further increased in chemoresistant cell lines compared with that in parental non-resistant cell lines (Figure 8C). Combining the clinicopathologic data from TCGA and TNMplot, and the IHC analysis from 74 patients, it was showed that the expression level of CDCA3 was aberrantly elevated in PC tumor tissues compared with that in the adjacent peritumoral tissues, and was negatively associated with patient survival (Figures 8D–H). Additionally, Pearson correlation analysis based on the clinicopathological data of 74 patients showed that the expression of CDCA3 and miR-188-5p was negatively correlated in PC (Figure 8I). To further clarify that circTNPO3 regulated CDCA3 through absorbing miR-188-5p, the expression level of CDCA3 was detected after co-transfection with sh-circTNPO3-1 and miR-188-5p inhibitor in BxPC-3/GR and CFPAC-1/GR cells. As shown in Figures 8J,K, transfection with sh-circTNPO3-1 attenuated CDCA3 expression, but this trend was rescued by miR-188-5p inhibitor.

### 3.5 CDCA3 is involved in NF-κB-p65 induced PC chemoresistance through regulating TRAF2

In our previous research, it was found that the tumorigenesis and GEM chemoresistance of PC might be associated with the abnormal activation of NF-κB signaling pathway (Ji et al., 2020). Through analyzing bioinformatics database, TRAF2 that was related with NF-κB signaling pathway and had protein interaction with CDCA3 was identified as the key mediator (Supplementary Figure S1D). Meanwhile, as shown in Figure 8L, Supplementarys Figures S1D–F, Pearson correlation analysis indicated that the expression level of CDCA3 and RELA (scilicet NF-κB-p65) was positively correlated with TRAF2. In order to explore the regulatory effect of CDCA3 on the expression level of TRAF2 and translocation of p65 from cytoplasm to nucleus, sh-CDCA3-1/2 used to silence CDCA3 was synthesized. After transfection with sh-CDCA3-1/2 in BxPC-3/GR and CFPAC-1/GR cells, the knockdown efficiency was detected by immunoblotting assay, finding that sh-CDCA3-2 might be a more efficient sequence (Figures 8M,N). Nucleoplasmic separation combined with immunoblotting experiments showed that compared with parental non-resistant BxPC-3 cells, the expression of CDCA3 and TRAF2 was higher in chemoresistant BxPC-3/GR cells, and the translocation of p65 from cytoplasm to nucleus was more significant (Figure 8O). Contrastively, sh-CDCA3-2 transfection obviously downregulated CDCA3 and TRAF2 expression, and attenuated p65 nuclear translocation tendency (Figure 8O). Meanwhile, as shown in Figure 8P, immunofluorescence assay more intuitively demonstrated the translocation of p65 (stained using red fluorescence) from cytoplasm to nucleus was stirred up by chemoresistance and was suspended by CDCA3 knockdown in BxPC-3/GR cells.

More importantly, in order to further clarify that circTNPO3 ultimately regulated the malignant biological characteristics and chemoresistance of PC through NF-κB signaling pathway, the overexpression or silence of circTNPO3 was performed in the parental non-resistant BxPC-3 cells and resistant BxPC-3/GR cells. As shown in Supplementary Figure S1G, circTNPO3 overexpression promoted the translocation of NF-κB-p65 from cytoplasm to nucleus in BxPC-3 cells. By contrast, silencing circTNPO3 attenuated the activation of the NF-κB in BxPC-3/GR cells (Supplementary Figure S1H).

### 3.6 CircTNPO3 might be developed as a novel therapeutic target for PC

Deoxyelephantopin (DET), a kind of small molecule compound, was extracted from the Chinese herbal medicine *E. scaber L.*. In our previous research, DET had been proven to possess noticeable antitumor effect in PC (Ji et al., 2022). In order to explore the potential of circTNPO3 as a therapeutic target and further reveal the antitumor mechanism of DET in PC, DET was applied for the subsequent experiments. The specific molecular structure of DET was listed in Supplementary Figure S2A. The qRT-PCR results found that the expression of circTNPO3 was significantly downregulated after DET treatment at a concentration of 40 μM for 24 h (Supplementary Figure S2B). In CCK-8 and EdU staining assays, DET decreased the proliferation ability of CFPAC-1 cells compared with that in the control group, conversely, overexpression of circTNPO3 partially attenuated the cytotoxicity of DET (Supplementary Figures S2C,D). Similar results were further confirmed in Transwell assays, finding that the migration and invasion abilities of CFPAC-1 cells were suppressed by DET treatment, but reversed by circTNPO3 overexpression (Supplementary Figure S2E). In order to further investigate the feasibility of DET in the treatment of PC, xenograft tumor model was conducted by subcutaneous injecting normal or transfected CFPAC-1 cells. As shown in Supplementary Figures S2F–I, DET exhibited inhibitory effects on the growth of PC (*n* = 5), however, overexpression of circTNPO3 attenuated the cytotoxicity of DET. Furthermore, the carcinogenic effect of circTNPO3 on PC was also confirmed *in vivo* experiment indirectly. Our current studies suggested that the disincentive efficacy of DET on the malignant phenotype of PC was partially depended on downregulating the expression of oncogene circTNPO3. Meanwhile, the potential of circTNPO3 as a novel therapeutic target for PC could also be inferred.

## 4 Discussions

PC, approximately 90% of which originates from pancreatic ductal epithelial, ranks as the sixth leading cause of cancer related death in both male and female worldwide (Stoffel et al., 2023). With the continuous innovation of medical technology, the prognosis of many tumors which could be diagnosed and intervened at early stage is substantially improving. However, due to the occult characteristics of PC, the vast majority of patients could not receive definitive diagnosis until they are at the advanced stages and miss out on the radical resection operation (Dudley and Brand, 2022). Meanwhile, hypovacular supply and dense tumor stroma of PC lead to inefficiencies in drug delivery and chemoresistance (Sherman and Beatty, 2023). In the field of immunotherapy, due to the complex signaling pathways exist in the tumor microenvironment, effector T lymphocytes could not be effectively recruited to PC foci, so this extremely lethal malignancy is also defined as an immunologically “cold” tumor and congenitally resistant to immune checkpoint inhibitors (Farhangnia et al., 2024). Considering the current status of PC, teasing out the principal pathogenesis of PC might provide important theoretical foundation for early diagnosis and therapy. In this research, the circTNPO3 was selected and defined as a dormant oncogene through comparing the differential expression between PC cells and tissues, and investigating the effect of circTNPO3 on PC malignant biological behaviors. Although there are some limitations in this research, such as the effect of tumor burden on circTNPO3 expression level could not be assessed due to the lack of preoperative and postoperative serum samples, and the absence of transgenic animal models also made it difficult to fully simulate the effects of circTNPO3 on the occurrence of PC *in vitro*, the circTNPO3/miR-188-5p/CDCA3/TRAF2/NF-κB axis in PC occurrence and progression is first reported, and still possesses theoretical significance.

Circular RNA (circRNA), a unique loop structure ncRNA, forms by backsplicing mode splicing 3′donor site directly with 5′acceptor site at the same exon (Drula et al., 2024). This nearly perfect loop domain invests circRNA with physicochemical hyperstability, combined with the diversity of circRNA variants and the regulation based on complementary base pairing criterion, the importance of circRNA in the research fields of human diseases, especially cancer, has become increasingly prominent (Chen et al., 2022). According to spatiotemporal expression and relative abundance, circRNA might participate in phenotype regulation at multiple stages of cancer (Chen et al., 2022). As one of the recently founded circRNAs, circTNPO3 is derived from the chromosome region of *TNPO3* gene (Xia et al., 2020). With the rapid development of the sequencing technology, PCR and primer design, the regulatory mechanism of circTNPO3 in human malignant tumors has been successively explored. It was found that circTNPO3 was highly expressed in resistant ovarian cancer cell lines and tissues, and facilitated paclitaxel chemoresistance via miR-1299/NEK2 axis (Xia et al., 2020). Yu et al. reported that the expression of circTNPO3 was lower in GC, and it might act as a tumor suppressor gene to inhibit GC metastasis through absorbing IGF2BP3 to downregulate the stability of MYC and SNAIL (Yu et al., 2021). A similar functional mechanism was founded in clear cell renal cell carcinoma, suggesting that circTNPO3 might downregulate the stability of SERPINH1 mRNA, a type of oncogene, by competitively binding to IGF2BP2 (Pan et al., 2022). The most recent research from Liu and colleagues found that aberrantly high expression of circTNPO3 might exacerbate the malignant biological behavior of hepatocellular carcinoma via classical ceRNA mechanism, circTNPO3/miR-199b-5p/STRN axis (Liu and Liu, 2023). The scarce four studies and the bidirectional characteristics of tumor promotion or inhibition make the regulatory role of circTNPO3 in human malignancies extremely mysterious and urgently need to be revealed. In our current research, the effects of circTNPO3 on PC growth, metastasis, apoptosis and chemoresistance were reported for the first time. More innovatively, the significance of the therapeutic target of circTNPO3 was also validated by treatment with deoxyelephantopin, a novel small molecule antitumor monomer from traditional Chinese medicine.

Gemcitabine (GEM), a kind of cytosine nucleoside derivatives, is explored and approved for the treatment of PC in the late 1990s (Beutel and Halbrook, 2023). Taking into account the tolerance especially in Asian populations, and the final benefits of other chemotherapy regimens such as FOLFIRINOX, modified FOLFIRINOX (mFOLFIRINOX) and NALIRIFOX (liposomal Irinotecan, Fluorouracil, Leucovorin, Oxaliplatin), gemcitabine-based chemotherapy regimens such as AG (Nab-paclitaxel, Gemcitabine), GS (Gemcitabine, Tegafur, Gimeracil, Oteracil) are still the first-line regimen for advanced PC, despite the existence of chemoresistance and adverse reactions (Nichetti et al., 2024). Compared with the chemotherapy side-effects which have more remedy to prevent, chemotherapy resistance of GEM, including primary and secondary resistance, has become a thornier issue in PC (Zhang T. et al., 2023). In this study, the regulatory effect of circTNPO3 on GEM chemoresistance was confirmed through establishing chemoresistant PC cell lines. Due to the deep anatomical location of PC, it is almost impossible to obtain chemoresistant PC pathological tissues by surgical excision or image-based guided puncture. Meanwhile, considering the instability of chemoresistant cells after injecting into experimental animal, chemoresistant animal model based on chemoresistant cells is still absent in our research. In order to accurately reveal the detailed mechanism of circTNPO3 regulating GEM chemoresistance in PC, the patient-derived xenograft (PDX) will be constructed in our future studies, and the chemoresistant animal models based on multigenerational stimulus of GEM might be applied successfully.

Cell division cycle-associated protein 3 (CDCA3), also referred to as gene-rich cluster protein C8 (CRCC8), is identified as a key trigger factor of cell mitosis (Wang et al., 2024). Molecular biology research has showed that CDCA3, an important functional subunit of the S phase kinase-associated protein 1 (SKP1)/Cullin/F-box E3 ubiquitin ligase protein complex, involved in the biodegradation of mitosis suppression associated tyrosine kinase WEE1 (Wang et al., 2024). Cumulative studies indicate that CDCA3 is abnormally high expressed in multiple human malignancies, such as glioma (Han et al., 2024), prostate cancer (Gu et al., 2023), pan-renal cell carcinoma (Luo et al., 2024), and bladder cancer (Shen et al., 2025), which may contribute to tumor progression as an oncogene. So far, there have been only one report about the regulatory effect of CDCA3 in PC studies. It should be noted that this study only analyzed the differential expression of CDCA3, and verified the oncogenic role of CDCA3 in PC through limited amount of cell biology experiments *in vivo* (Zou et al., 2020). In the present study, bioinformatics analysis was performed to elucidate the upstream and downstream networks of CDCA3, and a variety of experimental methods, including molecular biology experiments and animal experiments were used to support this novel regulatory circTNPO3/miR188-5p/CDCA3 axis. Significantly, by combining our previous research with the construction of chemoresistant PC cell lines, the perplexing protein interactions between CDCA3 and NF-κB a cancer-related signaling pathway were illustrated in present study, that is CDCA3/TRAF2/NF-κB axis.

## 5 Conclusion

**FIGURE 9:**
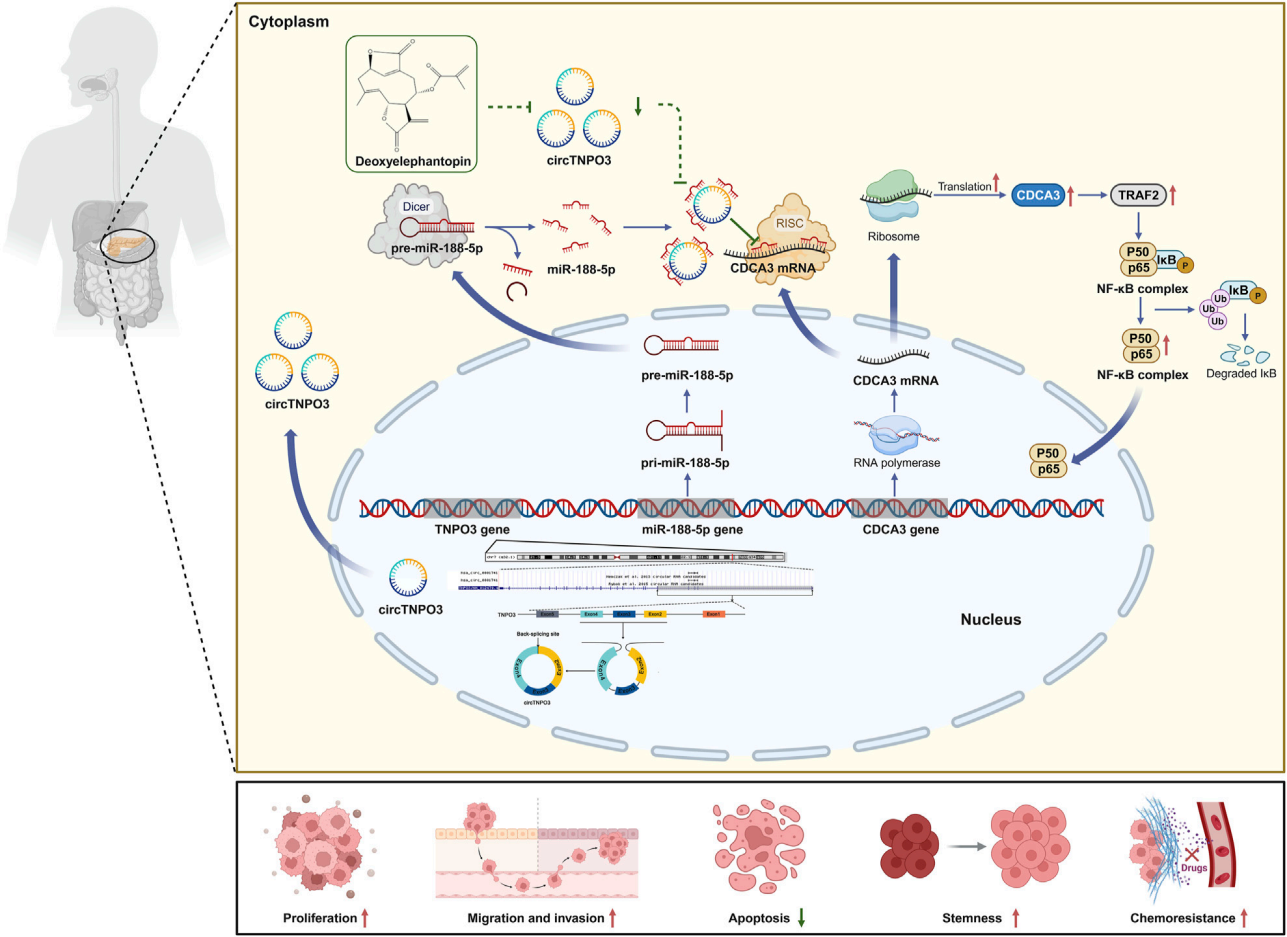
The mechanism of circTNPO3 regulating the malignant phenotype of PC.

In conclusion, the present study illustrates that circTNPO3 might promote the occurrence, progression and chemotherapy resistance of PC by miR-188-5p/CDCA3/TRAF2/NF-κB axis *in vivo and in vitro* (Figure 9). Therapeutically, as a novel small molecule antitumor monomer derived from traditional Chinese medicine, deoxyelephantopin might inhibit the malignant biological behavior and improve chemoresistance of PC through intervening the signaling axis led by circTNPO3 (Figure 9). Our study may lay a theoretical foundation for unveiling the core pathogenesis of PC, and provide new ideas for the development of novel early detection biomarkers, therapeutic targets and antitumor agents.

## Data Availability

The datasets presented in this study can be found in online repositories. The names of the repository/repositories and accession number(s) can be found in the article/Supplementary Material.

## Supplementary material

SUPPLEMENTARY FIGURE S1 The interaction networks between CDCA3, TRAF2 and RELA (NF-κB-p65). **(A)** The expression of linear TNPO3 after transfection with si-circTNPO3-1/2 was determined using immunoblotting assay. **(B)** The expression of circTNPO3 in HPDE6-C7 cells after circTNPO3 silence was detected using qRT-PCR. **(C)** The viability of HPDE6-C7 cells after circTNPO3 silence was analyzed using CCK-8 assay. **(D)** The interaction networks between CDCA3, TRAF2 and RELA (NF-κB-p65) were predicted and visualized using GeneMANIA database. **(E,F)** The correlation between CDCA3, TRAF2 and RELA (NF-κB-p65) in PC was analyzed through Pearson correlation coefficient. **(G)** The NF-κB-p65 nuclear translocation in BxPC-3 after circTNPO3 overexpression was evaluated using IF assay. **(H)** The NF-κB-p65 nuclear translocation in BxPC-3/GR cells after circTNPO3 knockdown was detected using IF assay. ***P* < 0.01. IF, immunofluorescence.

SUPPLEMENTARY FIGURE S2 The potential of circTNPO3 as a therapeutic target for PC. **(A)** The molecular structure of DET. **(B)** The expression of circTNPO3 in CFPAC-1 cells after treatment with DET was analyzed using qRT-PCR. ***P* < 0.01. **(C)** The viability of CFPAC-1 cells after treatment with DET was tested using CCK-8 assay. **(D)** The EdU positive staining representing proliferation of CFPAC-1 cells after circTNPO3 overexpression combined with DET treatment was quantified by EdU assay. **(E)** The migration and invasion of CFPAC-1 cells after circTNPO3 overexpression combined with DET treatment were detected using Transwell assay. **(F)** The detailed grouping and intervention strategies of the *in vivo* experiments. (*n* = 5). **(G,H)** Tumor growth curves were plotted according to the volume recorded every 3 days. **(I)** The final weight of the subcutaneous tumor. ***P* < 0.01 versus Empty vector group. #*P* < 0.05, ##*P* < 0.01 versus Empty vector combined with DET treatment group. CTL, control. DET, deoxyelephantopin.
