# Supplementary figures and images for: The functional roles of deoxyelephantopin potential target circTNPO3 in regulating pancreatic cancer malignant phenotype and gemcitabine chemoresistance via miR-188-5p/CDCA3/TRAF2-mediated remodeling of NF-κB signaling pathway
Source: Front Pharmacol. 2025 Jul 31;16:1613560. doi: 10.3389/fphar.2025.1613560 (PMC12350284; doi:10.3389/fphar.2025.1613560)

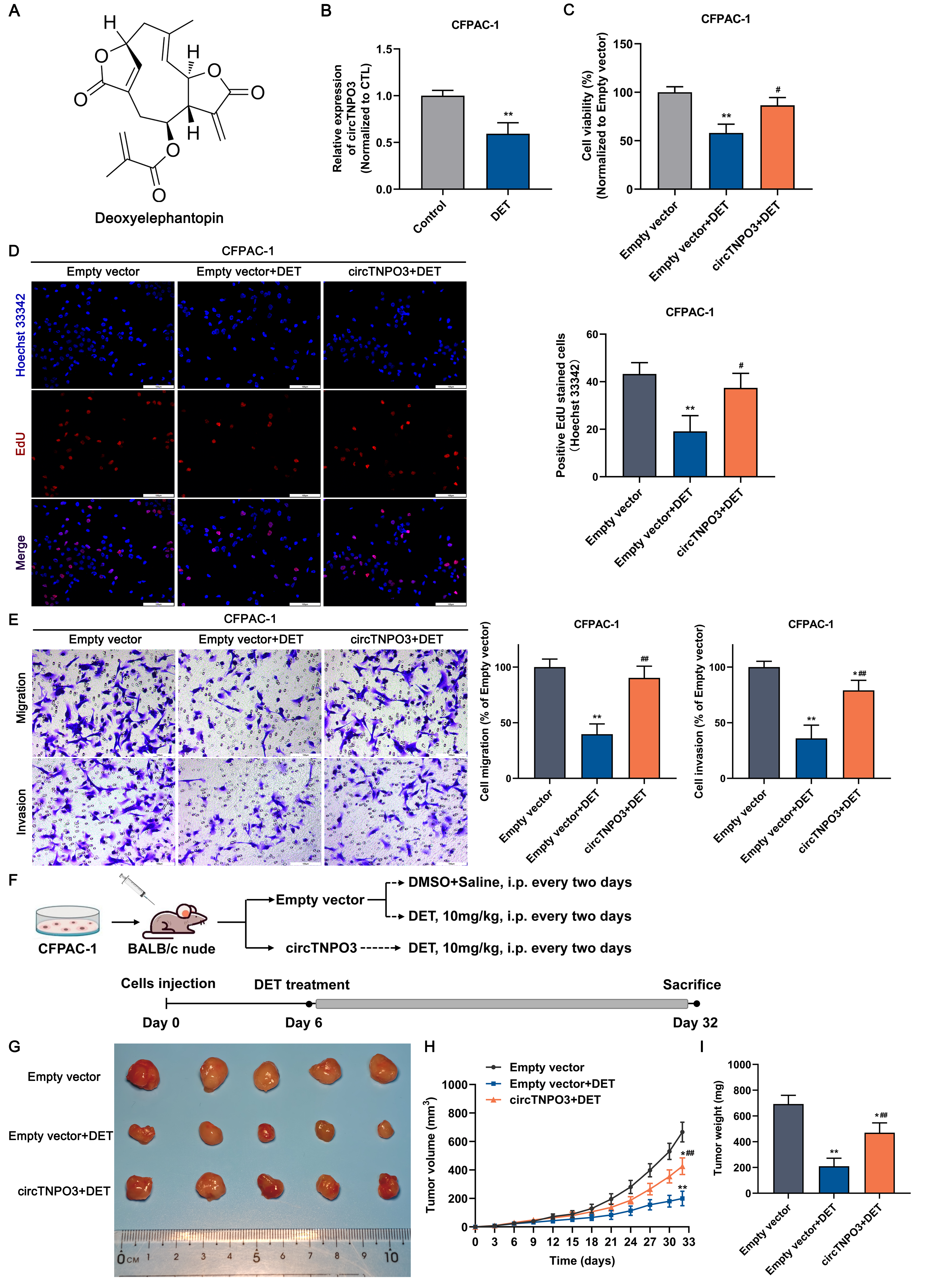

Supplement: Supplementary file 1 [file Image2.tif]

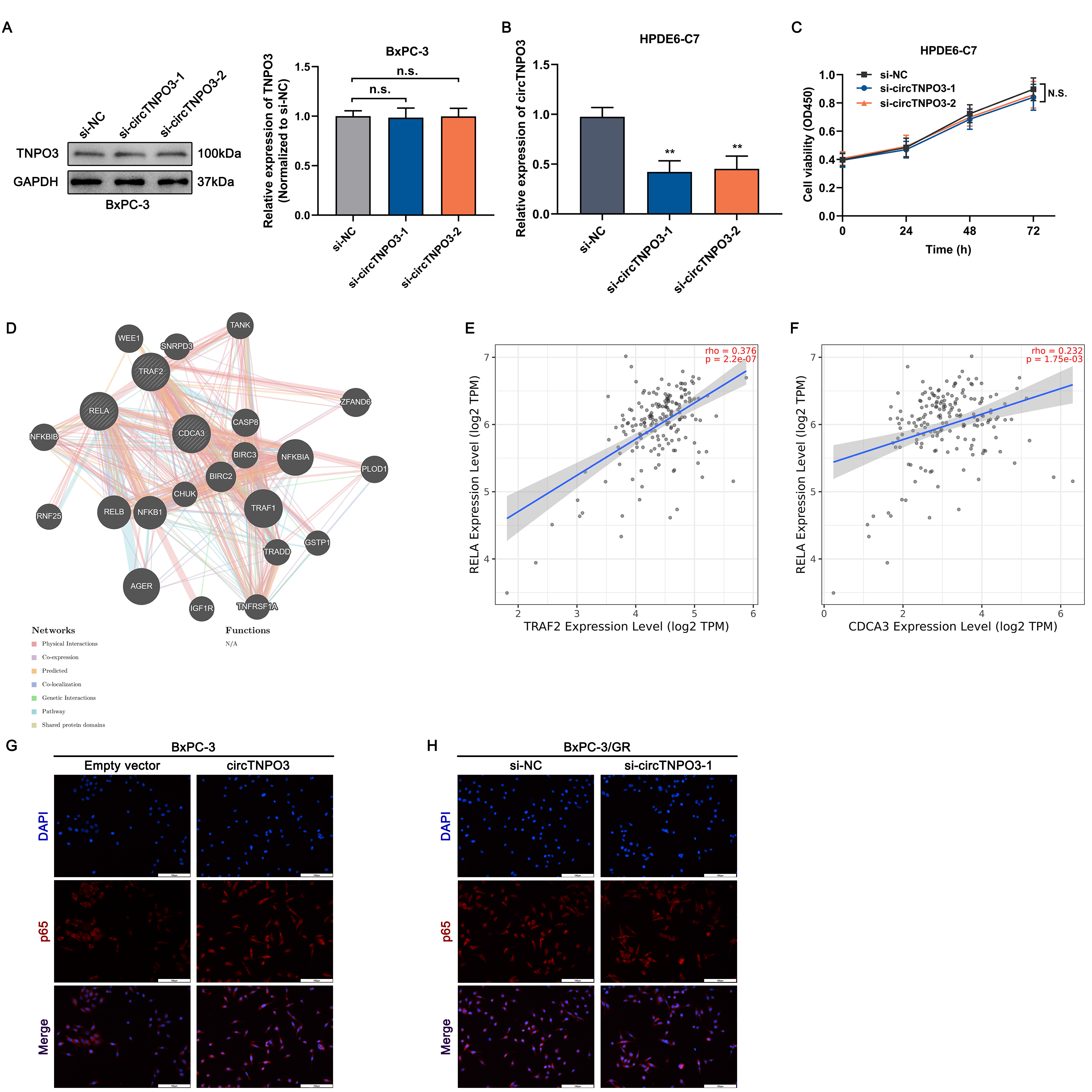

Supplement: Supplementary file 2 [file Image1.tif]
