# Supplementary material for: The functional roles of deoxyelephantopin potential target circTNPO3 in regulating pancreatic cancer malignant phenotype and gemcitabine chemoresistance via miR-188-5p/CDCA3/TRAF2-mediated remodeling of NF-κB signaling pathway
Source: Front Pharmacol. 2025 Jul 31;16:1613560. doi: 10.3389/fphar.2025.1613560 (PMC12350284; doi:10.3389/fphar.2025.1613560)
Supplement: Supplementary file 3 [file Table1.doc]

**Supplementary Table 1. The primer sequences used for qRT-PCR**

| **Target gene** | | **Primers (5′-3′)** |
| --- | --- | --- |
| circTNPO3 | 5′- GTCGTTCCTTACGAATTGGAGCT-3′ (Forward) | |
|  | 5′- GGTCTGTGCAGCAAAATAGCATG-3′ (Reverse) | |
| TNPO3 | 5′- TGGGAGATCTCAGACCAGTTGTTA-3′ (Forward) | |
|  | 5′- GGTGACAAGTCTTTCAAGTTCTGG-3′ (Reverse) | |
| miR-188-5p | 5′-CTCAACTGGTGTCGTGGAGTCGGCAATTCAGTTGAGCCCTCCAC-3′ (Forward) | |
|  | 5′-ACACTCCAGCTGGGCATCCCTTGCATGGTGG-3′ (Reverse) | |
| miR-199a-5p | 5′- CGCGCCCAGTGTTCAGACTAC-3′ (Forward) | |
|  | 5′- AGTGCAGGGTCCGAGGTATT-3′ (Reverse) | |
| miR-199b-5p | 5′-GCCGAGCCCAGTGTTTAGACTAT-3′ (Forward) | |
|  | 5′-GTCGTATCCAGTGCAGGGTCCGAGGTATTCGCACTGGATACGAC GAACAG-3′ (Reverse) | |
| miR-552-3p | 5′-GGTGACTGGTTAGACAAGTCGT-3′ (Forward) | |
|  | 5′-TGTCGTGGAGTCGGCAATTG-3′ (Reverse) | |
| CDCA3 | 5′- CACGGACACCTATGAAGACCA -3′ (Forward) | |
|  | 5′- GTTTGGAGGGGAACTCAGTCT-3′ (Reverse) | |
| GAPDH | 5′-TCCCATCACCATCTTCCA-3′ (Forward) | |
|  | 5′-CATCACGCCACAGTTTCC-3′ (Reverse) | |
| U6 | 5′-ATTGGAACGATACAGAGAAGATT3′ (Forward) | |
|  | 5′-GGAACGCTTCACGAATTTG-3′ (Reverse) | |

GAPDH, glyceraldehyde-3-phosphate dehydrogenase.
